# Health-related quality of life trajectories and loss of independence among community-dwelling older adults

**DOI:** 10.1007/s11357-025-01804-5

**Published:** 2025-07-29

**Authors:** Atsushi Takayama, Kenji Omae, Takeshima Taro, Takashi Yoshioka, Hiroaki Nakagawa, Ozaka Akihiro, Shunichi Fukuhara, The Sukagawa study group, Shunichi Fukuhara, Sugihiro Hamaguchi, Takao Tsuchiya, Mitsuru Munakata

**Affiliations:** 1 Department of Pharmacoepidemiology, Graduate School of Medicine and Public Health, Kyoto University, Yoshidakonoe-Cho, Sakyo-Ku, Kyoto, 606-8501 Japan; 2 Center for Innovative Research for Communities and Clinical Excellence (CiRC2LE), Fukushima Medical University, Fukushima City, Fukushima, Japan; 3 Department of Innovative Research and Education for Clinicians and Trainees (DiRECT), Fukushima Medical University Hospital, Fukushima City, Fukushima, Japan; 4 Center for University-Wide Education, School of Health and Social Services, Saitama Prefectural University, Koshigaya, Saitama Japan; 5 Department of Preventive Medicine and Public Health, School of Medicine, Keio University, Tokyo, Japan; 6 Institute of Clinical Epidemiology (iCE), Showa University, Tokyo, Japan; 7 Department of General Internal Medicine, Fukushima Medical University, Fukushima, Japan; 8 Department of General Medicine, Shirakawa Satellite for Teaching And Research (STAR), Fukushima Medical University, Fukushima, Japan; 9 Department of Health Policy and Management, Johns Hopkins Bloomberg School of Public Health (JHSPH), Baltimore, MD USA

**Keywords:** Aged, Functional status, Health-related quality of life, Healthy aging, Independent living

## Abstract

Health-related quality of life (HRQOL) is a key determinant of loss of independence (LOI) among older adults. However, the relationship between late-life HRQOL trajectory patterns and the risk of LOI remains unclear. This study utilized 5-year longitudinal data from the Sukagawa Study, and group-based trajectory modeling. HRQOL trajectories were analyzed using the mental component scale (MCS) and physical component scale (PCS) from the SF-8. Hazard ratios (HRs) and 95% confidence intervals (CIs) for LOI or death were calculated using Cox proportional hazards regression. Among 4043 participants (median age 78.8 years, interquartile range [IQR] 75.8–82.5; 56% women), HRQOL change score trajectories were categorized as decline, stable, and increase for MCS (11.7%, 81.0%, 7.2%) and PCS (10.9%, 81.4%, 7.6%). Over a mean follow-up of 66.4 months (IQR 48.0–73.0), LOI or death incidence was 42.5 per 1000 person-years (95% CI 39.8–45.2). Compared to the stable group, MCS decline was associated with higher LOI or death risk (HR 1.51, 95% CI 1.04–2.18), and an increase was associated with lower risk (HR 0.26, 95% CI 0.11–0.57). PCS decline was linked to higher LOI risk (HR 1.66, 95% CI 1.17–2.37), while an increase showed no significant association (HR 1.07, 95% CI 0.61–1.89). Longitudinal HRQOL trajectory is closely related to LOI risk among community-dwelling older adults. A declining trajectory in physical and mental components is strongly associated with higher LOI risk, while an increasing trajectory in the mental component is possibly associated with lower risk.

**Graphical Abstract:**

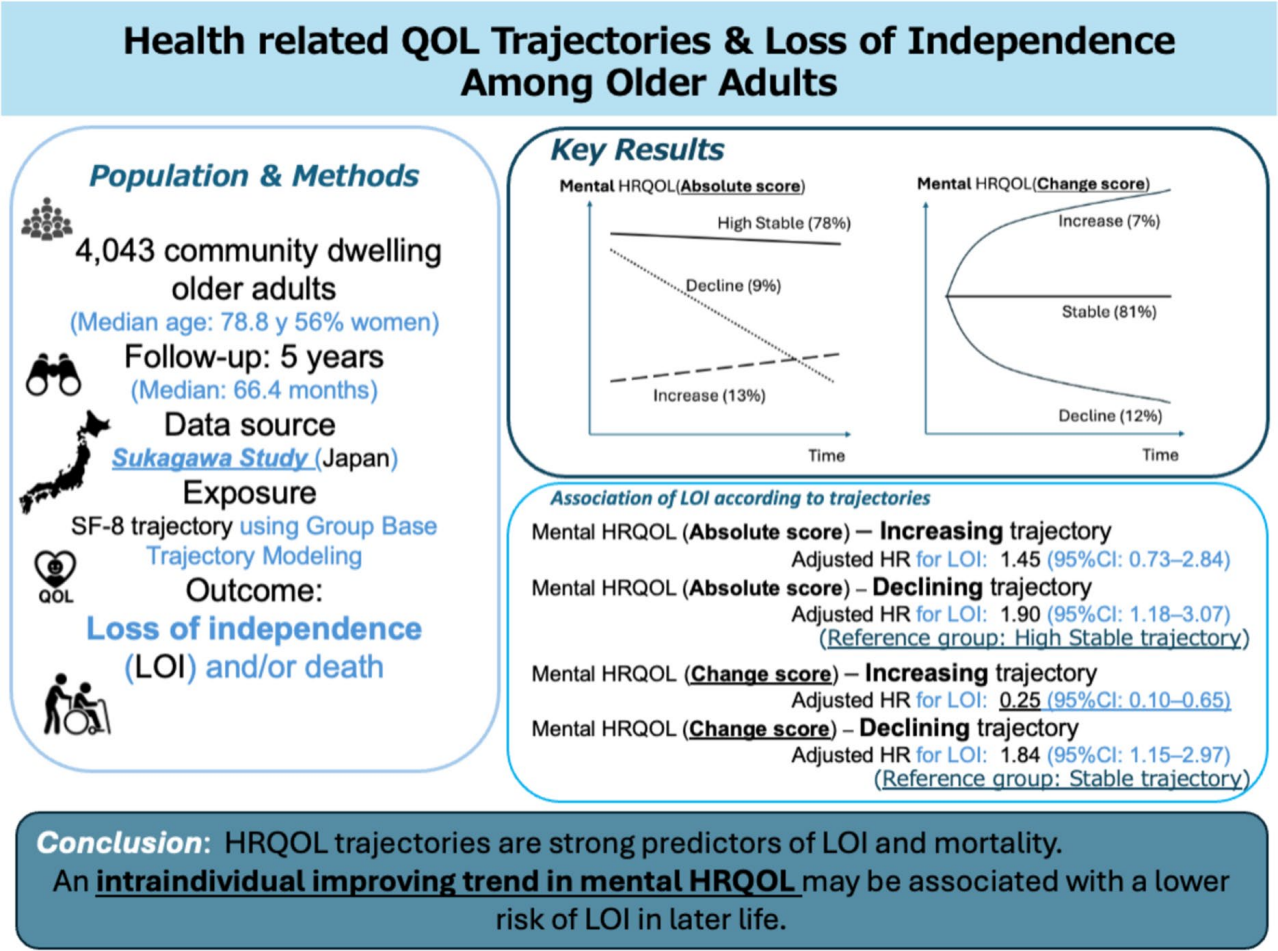

**Supplementary Information:**

The online version contains supplementary material available at 10.1007/s11357-025-01804-5.

## Introduction

The aging population constitutes an urgent global issue. The World Health Organization estimated that the global population ≥ 60 years will reach 1.4 and 2.1 billion by 2030 and 2050 [1]. To properly respond to this rapid global aging, understanding the process of healthy aging and focusing on expanding Healthy life expectancy (HLE) is getting more global attention [2–4].

Unfortunately, while life expectancy (LE) is improving in some countries, HLE can worsen [5]. The expansion of unhealthy life years—the gap between LE and HLE—can lead to excessive medical burdens and undesirable health outcomes. Therefore, extending HLE, rather than just LE, is one of the most urgent goals for aging societies. To maximize HLE, maintaining functional status and preventing LOI—both well-known determinants of HLE [6, 7]—should be prioritized as research focuses [8–10]. LOI is defined as the inability to make decisions and/or perform activities of daily living [8] and is strongly associated with aging, as is disability [11]. Previous studies have reported that HLE and its determinant, LOI, are strongly linked to quality of life and overall health status [2, 12, 13]. Consequently, improving quality of life, including health-related quality of life (HRQOL), among older adults is hypothesized to reduce the risk of LOI and represents a promising avenue for research.

However, there is currently no evidence to indicate whether HRQOL is practically modifiable in late life as part of the natural aging process. Furthermore, no evidence supports the hypothesis that improving HRQOL is associated with a reduced risk of losing independence, despite baseline HRQOL being a well-established determinant of mortality [14–17]. To address these gaps, we conducted a longitudinal assessment of HRQOL trajectory patterns among community-dwelling older adults and examined their association with the risk of losing independence.

## Methods

### Study design and setting

Data were derived from five waves of the Sukagawa Study surveys, conducted annually from 2017 to 2021 as part of an ongoing population-based cohort study of community-dwelling older adults in Sukagawa City. The Sukagawa Study is a collaborative effort between Sukagawa City, Iwase General Hospital, and Fukushima Medical University, with the objective of promoting healthy aging among the community’s senior population. The city’s demographic profile is similar to that of Japan. In 2017, Sukagawa City had a population of 76,625, of whom 20,633 (26.9%) were aged 65 years or older, and 10,128 (13.2%) were aged 75 years or older. For comparison, the proportion of older adults aged 65 and older in Japan as a whole was 27.7%, and those aged 75 and older accounted for 12.3%. Further details of the Sukagawa Study have been published elsewhere [18]. This study adhered to the principles of the Declaration of Helsinki and was approved by the Research Ethics Committee of Fukushima Medical University School of Medicine (registered approval number 2975). All participants provided written informed consent at the time of their cohort enrollment.

### Participant

Participants were community-dwelling older adults aged ≥ 75 years with a degree of independence, defined by a care level of ≤ 2 on the Long-Term Care Insurance system (LTCI) [19, 20]. The LTCI, a mandatory social insurance program in Japan, assigns care level 2 to individuals requiring minimal assistance with basic daily activities, such as bathing, medication management, or financial tasks [19, 20]. This threshold has been widely used in previous research as a marker for independent living [21, 22]. Only participants who responded to the survey at least three times and had no missing HRQOL data were included in the study.

### Outcome variable

The primary outcome was a composite of loss of independence (LOI) and all-cause death. Loss of the ability to live independently is one of the most critical outcomes for older adults [10]. LOI was defined as receiving care levels 3 to 5 under the LTCI certification system, which corresponds to requiring complete support for basic activities of daily living [19, 21, 22]. Since LTCI certification status determines the level of long-term care services, it is routinely and promptly assessed as needed through evaluations conducted by both an authorized care manager and the participant’s primary physician using standardized questionnaires. These evaluations were further processed by a computer-based system that estimates the time required for care. Final certification decisions were made by a board comprising various healthcare professionals [19, 23]. All outcome-related data were highly reliable, as they were provided by the municipal government based on annually reported administrative data. Moreover, these data were collected independently of participants’ responses to the survey questionnaire, ensuring comprehensive data coverage.

### Exposure variable

The exposure was the HRQOL trajectory class over 5 years, derived in advance through group-based trajectory analysis. We evaluated HRQOL trajectories using both the absolute scores of the Mental Component Summary (MCS) and Physical Component Summary (PCS) in the SF-8, as well as the change scores from baseline. Change scores were utilized to focus on and evaluate individual changes in HRQOL over time. HRQOL was assessed annually through a paper-based questionnaire using the SF-8 [24]. The Japanese version of the SF-8 has been validated [25] and is widely used to measure HRQOL among older populations [26–28]. Questionnaires were exhaustively distributed each year to all eligible residents within the municipality. Personnel from the municipal department responsible for the survey provided direct or telephone support to participants encountering difficulties in completing the questionnaire. Personalized reminders were also dispatched to non-respondents to maintain a high follow-up rate.

### HRQOL trajectory classification using the latent trajectory modeling

We used group-based trajectory modeling (GBTM) to identify patterns of HRQOL trajectories over the past 5 years. GBTM simplifies heterogeneous populations into more homogeneous clusters based on the similarity of trajectories (patterns of change) by modeling between-person differences in within-person changes using longitudinal observational data [29–31]. We modeled absolute scores of MCS and PCS and their changes from baseline separately. First, we sequentially generated models with the number of trajectory groups ranging from two to seven, using polynomial functions from first to third order. We assessed model appropriateness using the following criteria: an average posterior probability of assignments for each trajectory > 0.7; odds of correct classification for each trajectory > 5; and a minimum of 5% of the total population assigned to each trajectory group [29, 30]. Based on the Bayesian information criterion (BIC) and the clinical interpretability of the number and shape of the trajectories, we identified the best-fit number of classes. We evaluated the model fitness between the fixed-effect homoscedastic, random-effects homoscedastic across classes, and class-specific random-effect models, and plotted the final model of trajectories over time for each class. Supplementary Tables 1–4 present the results of the model selection process and the final trajectory models. We plotted individual observed trajectories of 100 randomly selected participants (spaghetti plots) to describe intraclass change patterns (Supplementary Figs. 1–4).

### Covariates

Based on previous studies and clinical perspectives, we considered the following: sex, age, body mass index (BMI), smoking habits (never, current, past), alcohol intake (never; 1–2, 3–4, or 5–6 days/per week; or daily), marital status, living alone, highest education level (elementary school, junior high school, high school, junior college, vocational school, university, or graduate school), annual household income < \3 million ($20,600), UCLA Loneliness Scale score ≥ 6, SARC-F ≥ 4, history of malignant disease, myocardial infarction, stroke, depression, and diabetes, and baseline MCS and PCS. All covariates were obtained from the cohort entry for each individual.

### Statistical analysis

Participant characteristics were summarized as frequencies and proportions (%) for categorical variables, and as medians and interquartile ranges (IQRs) for continuous variables. We calculated the person-time of follow-up for each participant from the month of cohort entry to the month of receiving LTCI care levels ≥ 3, death, relocation from the city, or the end of follow-up, whichever occurred first. Survival time data were presented using Kaplan–Meier survival curves. To quantitatively evaluate the association between HRQOL trajectories and outcomes, Cox proportional hazards regression was used to calculate adjusted hazard ratios (HRs) and 95% confidence intervals (CIs) for the composite outcome, as well as for LOI and all-cause mortality separately, using a complete case approach. In an exploratory secondary analysis, we simultaneously estimated the HRs (95% CIs) for the risk of outcomes across groups stratified by all combined classes of MCS and PCS trajectories, as well as by the combined change-score trajectories. All statistical analyses were performed using R, version 4.2.2.

### Sensitivity analysis

Two sensitivity analyses were conducted. First, to examine the predictive association between HRQOL trajectories and LOI, we repeated the main analysis, including only outcomes that occurred after 60 months (the trajectory assessment period), while censoring outcomes that occurred before 60 months. Second, we reperformed the main analysis among participants who were fully independent at baseline according to the LTCI.

## Results

### Participants’ characteristics

**Fig. 1:**
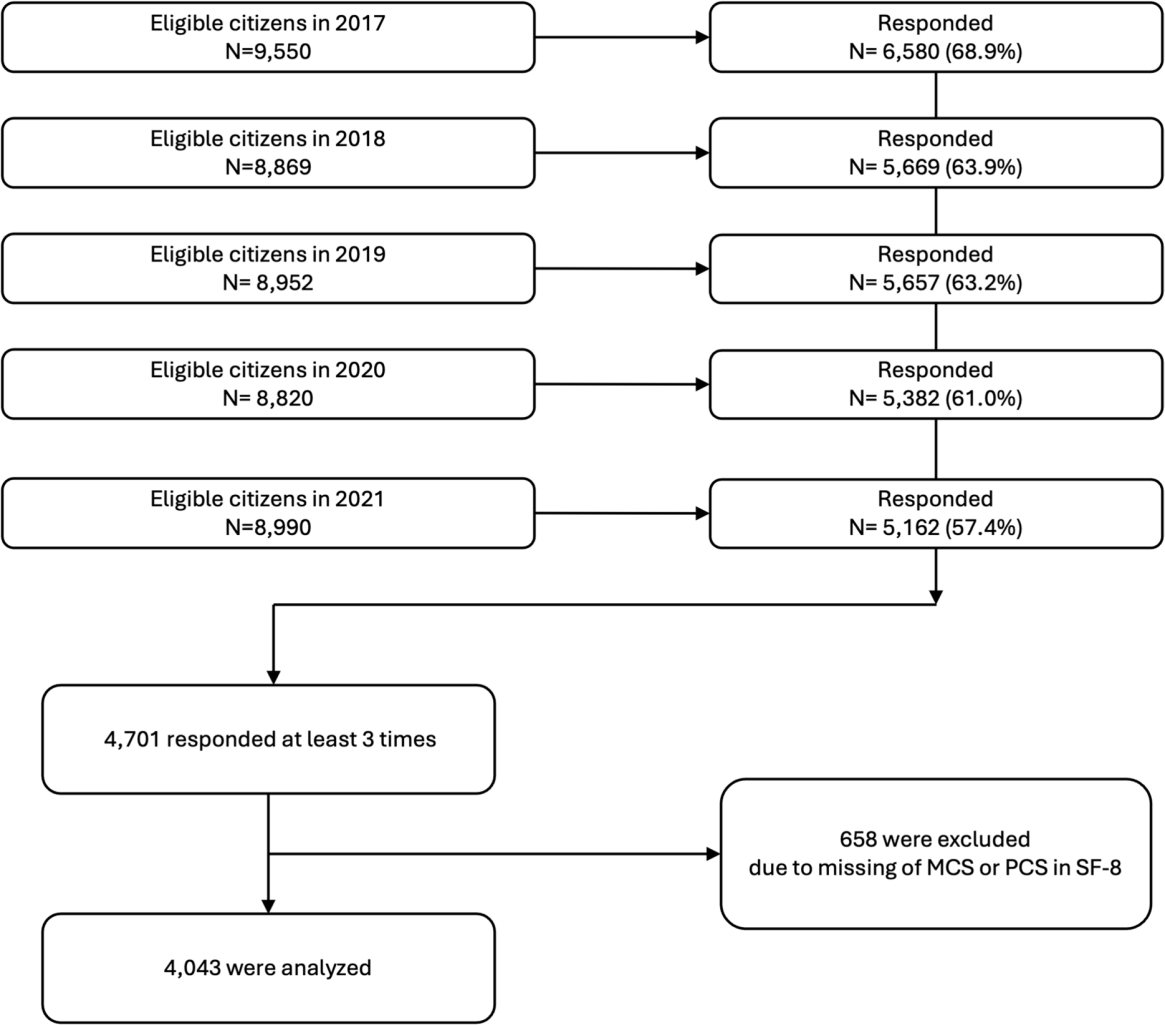
Study flow diagram. MCS, mental component scale; PCS, physical component scale

Figure 1 shows the study flow. From 2017 to 2021, annual respondents ranged from 5126 to 6580. Among them, 4701 individuals responded at least three times. After excluding 658 with missing SF-8 MCS or PCS data, 4043 participants were included in the final analysis. Table 1 shows the characteristics of the 4043 participants included in the analysis; a total of 4043 participants were included in the analysis. The median (IQR) age at baseline was 78.8 years (75.8–82.5), and 2254 participants (56%) were women. The median (IQR) MCS and PCS scores at baseline were 52.9 (48.4–55.0) and 50.0 (44.0–54.0), respectively. At baseline, 3777 participants (93%) were classified as independent. The characteristics and outcome occurrences of ineligible patients are presented in Supplemental Tables 5 and 6. No remarkable differences in baseline covariates were observed between eligible and ineligible participants; however, the incidence of outcomes was relatively higher among ineligible participants.

**Table 1:** Baseline characteristics of the participants

Characteristics	*N* = 4043
Age (years), median (IQR)	78.8 (75.8, 82.5)
Sex, *n* (%)	
Men	1789 (44%)
Women	2254 (56%)
BMI (kg/m^2^), median (IQR)	22.9 (20.8, 25.1)
Smoking habit, *n* (%)	
Never	2499 (63%)
Past	1261 (32%)
Current	220 (5.5%)
Missing	63
Alcohol habit, *n* (%)	
Never	2877 (72%)
1–2 days/week	254 (6.3%)
3–4 days/week	226 (5.6%)
5–6 days/week	155 (3.9%)
Everyday	497 (12%)
Missing	34
Unmarried, *n* (%)	31 (0.8%)
Living alone, *n* (%)	637 (16%)
Missing	92
Highest level of education, *n* (%)	
Elementary school	180 (4.9%)
Junior high school	1680 (46%)
High school	1199 (33%)
Junior college	85 (2.3%)
Vocational school	252 (6.8%)
University	230 (6.2%)
Graduate school	5 (0.1%)
Others	50 (1.4%)
Missing	362
Annual household income < ¥3 M ($20.6 K), *n* (%)	978 (24%)
UCLA loneliness scale ≥ 6, *n* (%)	607 (16%)
Missing	322
SARC-F ≥ 4, *n* (%)	1021 (28%)
Missing	384
History of malignant disease, *n* (%)	445 (11%)
History of myocardial infarction, *n* (%)	536 (13%)
History of stroke, *n* (%)	584 (14%)
History of depression, *n* (%)	591 (15%)
Diabetes, *n* (%)	499 (12%)
Baseline MCS score, median (IQR)	52.9 (48.4, 55.0)
Baseline PCS score, median (IQR)	50.0 (44, 54)
Baseline LTCI level, *n* (%)	
Independent	3777 (93%)
Support care level 1	105 (2.6%)
Support care level 2	161 (4.0%)
Number of times of response, *n* (%)	
3	947 (23%)
4	1593 (39%)
5	1503 (37%)

Continuous variables are described as median (IQR) and categorical variables are described as number (%). *BMI*: body mass index, *M*: million, *K*: thousand, *UCLA*: University of California, Los Angeles, *SARC-F*: a symptom score to predict persons with sarcopenia at risk for poor functional outcomes, *MCS*: mental component scale, *PCS*: physical component scale, *LTCI*: long-term care insurance. ¥3 M ($20.6 K) is mean annual household income among population aged ≥ 65 in Japan

### HRQOL trajectory grouping

**Fig. 2:**
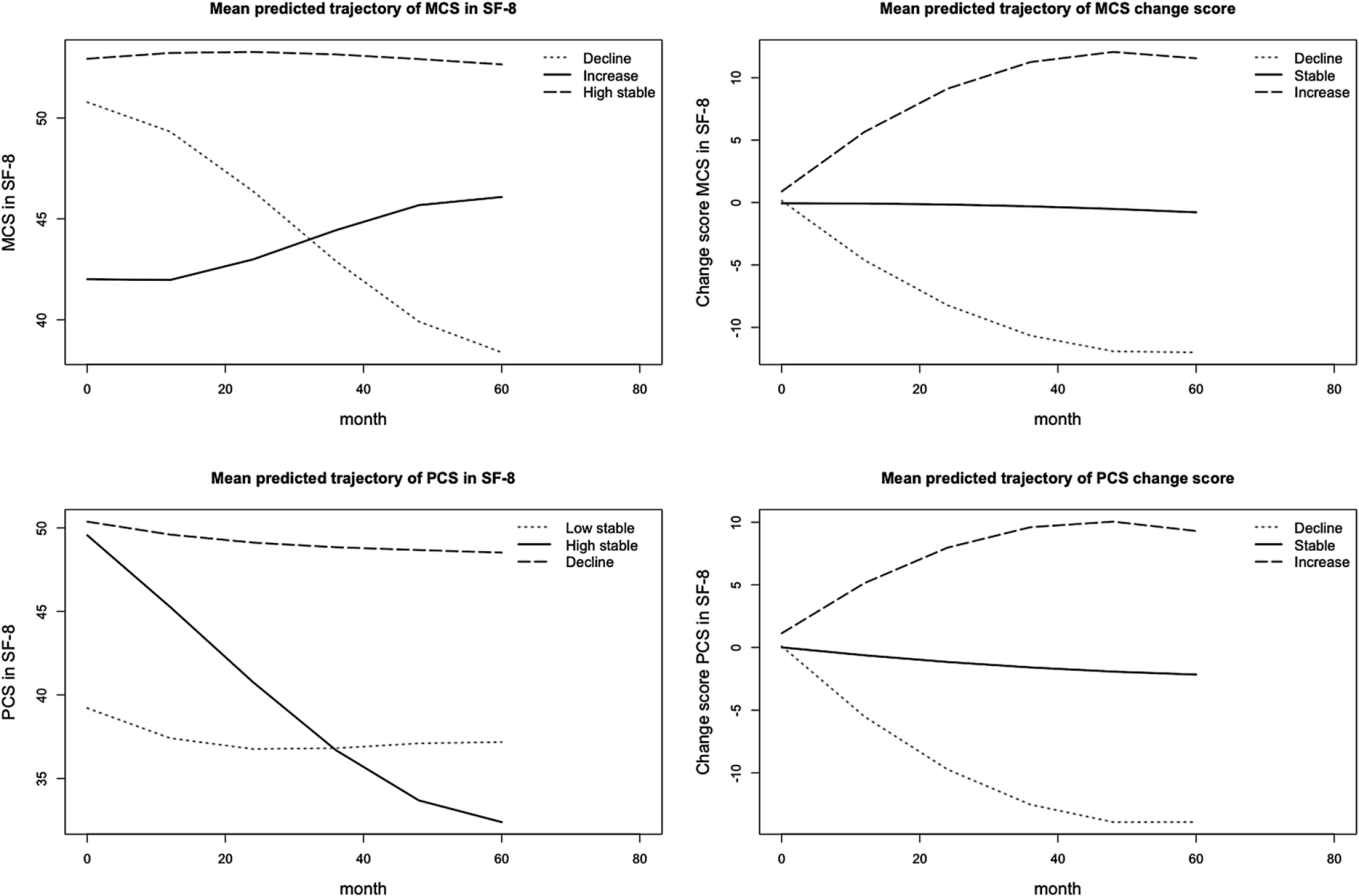
Trajectories of MCS, PCS, and change score of those MCS, mental component scale; PCS, physical component scale

Figure 2 illustrates the trajectories of MCS, PCS, and their change scores. MCS trajectory patterns were classified into three groups: decline (*n* = 376, 9.3%), high-stable (*n* = 3160, 78.2%), and increase (*n* = 507, 12.5%). PCS trajectory patterns were also categorized into three groups: low-stable (*n* = 434, 10.7%), high-stable (*n* = 3265, 80.8%), and decline (*n* = 344, 8.5%). For MCS change score trajectories, participants were grouped into decline (*n* = 475, 11.7%), stable (*n* = 3276, 81.0%), and increase (*n* = 292, 7.2%). Similarly, PCS change score trajectories were categorized as decline (*n* = 442, 10.9%), stable (*n* = 3292, 81.4%), and increase (*n* = 309, 7.6%). Supplementary Tables 7–10 present participants’ characteristics according to these trajectory classifications.

### Outcome occurrence

During the follow-up period, 850 composite outcome events were identified, comprising 518 cases of LOI and 522 deaths. The incidence rates (95% CI) per 1000 person-years for the composite outcome, LOI, and death were 42.5 (39.8–45.2), 25.9 (23.9–28.1), and 24.8 (22.8–26.8), respectively. The median (IQR) follow-up duration was 66.4 (48.0–73.0) months.

### Association of outcomes with HRQOL-component-stratified trajectories

Supplementary Figs. 5–8 show Kaplan–Meier survival estimates for the trajectory-stratified composite outcome–free survival. In both MCS and PCS, the decline group had shorter LOI-free survival. The MCS trajectories were not associated with the risk of composite outcome. However, the MCS trajectories were associated with LOI (Table 2). Relative to the high-stable group in MCS, the decline group was associated with a higher risk of LOI (adjusted HR, 1.90; 95% CI, 1.18–3.07). The PCS trajectories were associated with the risk of composite outcome, LOI, and death. Relative to the high-stable group in PCS, the decline group was associated with higher risks of composite outcome (adjusted HR, 1.68; 95% CI, 1.14–2.47), LOI (adjusted HR, 1.70; 95% CI, 1.04–2.77), and death (adjusted HR, 2.01; 95% CI, 1.22–3.33). The MCS change score trajectories were associated with the risk of composite outcome and LOI. Relative to the stable group in MCS change score, the decline group was associated with higher risks of composite outcome (adjusted HR, 1.51; 95% CI, 1.04–2.18) and LOI (adjusted HR, 1.84; 95% CI, 1.15–2.97); the increase group was associated with lower risks of composite outcome (adjusted HR, 0.26; 95% CI, 0.11–0.57) and LOI (adjusted HR, 0.25; 95% CI, 0.10–0.65). The PCS change score trajectories were associated with the risk of composite outcome and death. Relative to the stable group in PCS change score, the decline group was associated with higher risks of composite outcome (adjusted HR, 1.66; 95% CI, 1.17–2.37) and death (adjusted HR, 1.96; 95% CI, 1.26–3.06). However, compared with the stable group in PCS change score, the increase group in PCS change score was not associated with lower risks of composite outcome (adjusted HR, 1.07; 95% CI, 0.61–1.89) and LOI (adjusted HR, 1.22; 95% CI, 0.60–2.49) (Table 2).

**Table 2:** Association of outcomes according to trajectories of each component of HRQOL

	**MCS trajectory class**		
	Increase (*N* = 507)	High stable (*N* = 3160)	Decline (*N* = 376)
**Composite**			
Adjusted hazard ratio (95% CI)	1.36 (0.80–2.31)	1 [Reference]	1.42 (0.96–2.11)
**Loss of independence**			
Adjusted hazard ratio (95% CI)	1.45 (0.73–2.84)	1 [Reference]	1.90 (1.18–3.07)*
**Death**			
Adjusted hazard ratio (95% CI)	1.50 (0.74–3.03)	1 [Reference]	1.25 (0.72–2.16)
	PCS trajectory class		
	Low stable (*N* = 434)	High stable (*N* = 3265)	Decline (*N* = 344)
**Composite**			
Adjusted hazard ratio (95% CI)	1.24 (0.69–2.23)	1 [Reference]	1.68 (1.14–2.47)*
**Loss of independence**			
Adjusted hazard ratio (95% CI)	1.09 (0.51–2.34)	1 [Reference]	1.70 (1.04–2.77)*
**Death**			
Adjusted hazard ratio (95% CI)	2.05 (0.94–4.46)	1 [Reference]	2.01 (1.22–3.33)*
	MCS change score trajectory class		
	Decline (*N* = 475)	Stable (*N* = 3276)	Increase (*N* = 292)
**Composite**			
Adjusted hazard ratio (95% CI)	1.51 (1.04–2.18)*	1 [Reference]	0.26 (0.11–0.57)*
**Loss of independence**			
Adjusted hazard ratio (95% CI)	1.84 (1.15–2.97)*	1 [Reference]	0.25 (0.10–0.65)*
**Death**			
Adjusted hazard ratio (95% CI)	1.40 (0.86–2.28)	1 [Reference]	0.42 (0.16–1.13)
	PCS change score trajectory class		
	Decline (*N* = 442)	Stable (*N* = 3292)	Increase (*N* = 309)
**Composite**			
Adjusted hazard ratio (95% CI)	1.66 (1.17–2.37)*	1 [Reference]	1.07 (0.61–1.89)
**Loss of independence**			
Adjusted hazard ratio (95% CI)	1.52 (0.96–2.42)	1 [Reference]	1.22 (0.60–2.49)
**Death**			
Adjusted hazard ratio (95% CI)	1.96 (1.26–3.06)*	1 [Reference]	0.75 (0.30–1.86)

*95% CI* 95% confidence interval, *p value < 0.05. The composite outcome is defined as loss of independence (LOI) or death from any cause

Adjusted for sex, age, body mass index, smoking habits, alcohol intake, marital status, living alone, highest level of education, annual household income, UCLA loneliness scale ≥ 6, SARC-F ≥ 4, history of being diagnosed with malignant disease, myocardial infarction, stroke, depression, and diabetes, baseline MCS and PCS in SF-8

### Association of outcomes with nine MCS- and PCS (change score)-trajectory-stratified classes

Supplementary Figs. 9 and 10 show Kaplan–Meier survival estimates for MCS- and PCS-simultaneously stratified composite outcome–free survival, as well as those based on change scores. The MCS- and PCS-stratified trajectories were significantly associated with the risks of composite outcome, LOI, and death (Table 3). The decline trajectory in MCS and PCS (class 9), compared with the high-stable trajectory in MCS and PCS (class 1), was associated with higher risks of composite outcome (adjusted HR, 2.90; 95% CI, 1.73–4.87), LOI (adjusted HR, 3.76; 95% CI, 2.00–7.09), and death (adjusted HR, 2.68; 95% CI, 1.33–5.39). The MCS and PCS change-score trajectory-stratified nine groups were associated with the risks of composite outcome, LOI, and death. The decline trajectory in MCS and PCS change score (class 9), compared with the stable trajectory in MCS and PCS change score (class 1), was associated with higher risks of composite outcome (adjusted HR, 3.06; 95% CI, 1.84–5.09), LOI (adjusted HR, 3.76; 95% CI, 2.01–7.03), and death (adjusted HR, 3.13; 95% CI, 1.66–5.90). The increase trajectory in MCS change score and stable trajectory in PCS change score (class 4), compared with the stable trajectory in MCS and PCS change score (class 1), was associated with lower risks of composite outcome (adjusted HR, 0.11; 95% CI, 0.03–0.48), LOI (adjusted HR, 0.08; 95% CI, 0.01–0.59), and death (adjusted HR, 0.13; 95% CI, 0.02–0.96) (Table 3).

**Table 3:** Association of outcomes according to 9 classes stratified by MCS and PCS (change score) trajectories

					**9 classes stratified by MCS and PCS trajectories**				
	Class 1 M: high stable and P: high stable (*N* = 2753)	Class 2 M: high stable and P: low stable (*N* = 234)	Class 3 M: high stable and P: decline (*N* = 173)	Class 4 M: increase and P: high stable (*N* = 300)	Class 5 M: increase and P: low stable (*N* = 132)	Class 6 M: increase and P: decline (*N* = 75)	Class 7 M: decline and P: high stable (*N* = 212)	Class 8 M: decline and P: low stable (*N* = 68)	Class 9 M: decline and P: decline (*N* = 96)
**Composite**									
**aHR (95% CI)**	1 [Ref]	1.18 (0.56–2.51)	1.24 (0.70–2.18)	1.09 (0.54–2.19)	2.85 (1.14–7.11)*	1.39 (0.62–3.12)	1.08 (0.59–1.96)	0.68 (0.23–2.02)	2.90 (1.73–4.87)*
**Loss of independence**									
**aHR (95% CI)**	1 [Ref]	1.25 (0.48–3.22)	1.17 (0.53–2.54)	1.26 (0.54–2.95)	2.67 (0.84–8.53)	1.35 (0.48–3.78)	1.69 (0.86–3.33)	0.53 (0.11–2.42)	3.76 (2.00–7.09)*
**Death**									
**aHR (95% CI)**	1 [Ref]	1.32 (0.44–3.98)	1.57 (0.78–3.15)	0.84 (0.35–1.99)	1.16 (0.25–5.41)	2.01 (0.73–5.49)	0.84 (0.35–1.99)	1.16 (0.25–5.41)	2.68 (1.33–5.39)*
					9 classes stratified by MCS and PCS change score trajectories				
	Class 1 Mc: stable and Pc: stable (*N* = 2778)	Class 2 Mc: stable and Pc: increase (*N* = 204)	Class 3 Mc: stable and Pc: decline (*N* = 294)	Class 4 Mc: increase and Pc: stable (*N* = 199)	Class 5 Mc: increase and Pc: increase (*N* = 33)	Class 6 Mc: increase and Pc: decline (*N* = 60)	Class 7 Mc: decline and Pc: stable (*N* = 315)	Class 8 Mc: decline and Pc: increase (*N* = 72)	Class 9 Mc: decline and Pc: decline (*N* = 88)
**Composite**									
**aHR (95% CI)**	1 [Ref]	1.14 (0.61–2.15)	1.12 (0.71–1.77)	0.11 (0.03–0.48)*	0.37 (0.05–2.85)	0.77 (0.26–2.25)	1.05 (0.63–1.76)	0.99 (0.34–2.92)	3.06 (1.84–5.09)*
**Loss of independence**									
**aHR (95% CI)**	1 [Ref]	1.63 (0.77–3.46)	0.83 (0.43–1.59)	0.08 (0.01–0.59)*	0.45 (0.06–3.64)	0.76 (0.21–2.74)	1.41 (0.75–2.64)	0.42 (0.05–3.28)	3.76 (2.01–7.03)*
**Death**									
**aHR (95% CI)**	1 [Ref]	0.49 (0.15–1.65)	1.21 (0.67–2.17)	0.13 (0.02–0.96)*	NA	1.65 (0.53–5.13)	0.68 (0.32–1.45)	1.50 (0.42–5.41)	3.13 (1.66–5.90)*

*M* mental component scale, *P* physical component scale, *Mc* mental component scale change score, *Pc* physical component scale change score, *aHR* adjusted hazard ratio, *95% CI* 95% confidence interval, *p value < 0.05. The composite outcome is defined as loss of independence (LOI) or death from any cause. Adjusted for sex, age, body mass index, smoking habits, alcohol intake, marital status, living alone, highest level of education, annual household income, UCLA loneliness scale ≥ 6, SARC-F ≥ 4, history of being diagnosed with malignant disease, myocardial infarction, stroke, depression, and diabetes, baseline MCS and PCS in SF-8. *NA* not available (aHR not estimated due to insufficient events in the subgroup)

### Sensitivity analysis

The results of the sensitivity analyses were generally consistent with those of the main analyses. Supplementary Table 11 shows the association between trajectory patterns of each component of HRQOL and outcomes occurring after 60 months. The MCS trajectories were associated with the risk of composite outcome and LOI, all occurring after 60 months. Relative to the high-stable group in MCS, the decline group was associated with higher risks of composite outcome (adjusted HR, 1.63; 95% CI, 1.06–2.51), and LOI (adjusted HR, 2.15; 95% CI, 1.25–3.69), all occurring after 60 months. The PCS trajectories were associated with the risks of composite outcome, LOI, and death. Relative to the high-stable group in PCS, the decline group was associated with higher risks of composite outcome (adjusted HR, 2.32; 95% CI, 1.57–3.45), LOI (adjusted HR, 2.34; 95% CI, 1.38–3.96), and death (adjusted HR, 3.06; 95% CI, 1.94–4.82), all occurring after 60 months. The MCS change score trajectories were associated with the risks of composite outcome, LOI, and death, all occurring after 60 months. Relative to the stable group in MCS change scores, the increase group was associated with lower risks of composite outcome (adjusted HR, 0.32; 95% CI, 0.13–0.78), LOI (adjusted HR, 0.30; 95% CI, 0.10–0.91), and death (adjusted HR, 0.36; 95% CI, 0.14–0.98); and the decline group was associated with a higher risk of composite outcome (adjusted HR, 1.51; 95% CI, 1.00–2.28), all occurring after 60 months. The PCS change score trajectories were associated with the risks of composite outcome, LOI, and death. Relative to the stable group in PCS change score, the decline group was associated with higher risks of composite outcome (adjusted HR, 2.25; 95% CI, 1.57–3.23), LOI (adjusted HR, 2.11; 95% CI, 1.28–3.46), and death (adjusted HR, 2.58; 95% CI, 1.71–3.90), all occurring after 60 months.

Supplementary Table 12 shows the association of outcomes according to trajectories of each component of HRQOL among participants who were independent at baseline. The MCS trajectories were associated with the risks of composite outcome and LOI among the population. Relative to the high-stable group in MCS, the decline group was associated with higher risks of composite outcome (adjusted HR, 1.50; 95% CI, 1.00–2.25), and LOI (adjusted HR, 2.03; 95% CI, 1.24–3.33) among the independent population. The PCS trajectories were associated with the risks of composite outcome, LOI, and death. Relative to the high-stable group in PCS, the decline group was associated with higher risks of composite outcome (adjusted HR, 1.78; 95% CI, 1.19–2.66), LOI (adjusted HR, 1.87; 95% CI, 1.12–3.10), and death (adjusted HR, 1.98; 95% CI, 1.18–3.32). The MCS change score trajectories were associated with the risks of composite outcome and LOI among the population. Relative to the stable group in MCS change score, the increase group was associated with lower risks of composite outcome (adjusted HR, 0.20; 95% CI, 0.08–0.52), and LOI (adjusted HR, 0.23; 95% CI, 0.08–0.66), while the decline group was associated with a higher risk of LOI (adjusted HR, 1.73; 95% CI, 1.05–2.84) among participants who were independent at baseline. The PCS change score trajectories were associated with the risks of composite outcome, LOI, and death among the population. Relative to the stable group in PCS change score, the decline group was associated with higher risks of composite outcome (adjusted HR, 1.75; 95% CI, 1.21–2.51), LOI (adjusted HR, 1.64; 95% CI, 1.02–2.65), and death (adjusted HR, 1.93; 95% CI, 1.22–3.06) among participants who were independent at baseline.

## Discussion

### Key results

Among community-dwelling older adults in Japan, this population-based cohort study using 5-year HRQOL trajectories revealed associations between HRQOL trajectory patterns and LOI risk. The absolute score of the MCS trajectory revealed three patterns (increase, high-stable, and decline). The absolute score of the PCS trajectory also revealed three patterns (low-stable, high-stable, and decline). The decline group in the absolute score of PCS, compared with the high-stable group, was associated with higher risks of composite outcome, LOI, and death. Three patterns of MCS and PCS change score trajectories (decline, stable, and increase) also showed that the decline group was associated with higher risks of those outcomes. Notably, the increase in the MCS change-score trajectory was associated with a lower risk of LOI.

### Comparison with previous studies

A large amount of literature has concluded that HRQOL is a determinant factor in mortality [14–17] and LOI [28, 32, 33] among older adults but those studies utilized only a one-time baseline HRQOL. Several studies, however, evaluated the change or trajectory of HRQOL of older adults in various contexts [34–38]. Philip et al. classified three trajectory groups (low, moderate, high) for 10 years of MCS and PCS in SF-36 among Royal Canadian Air Force aircrew veterans with a mean age of 85.5 [39]. They did not show the shapes of trajectories but the robust association between low-HRQOL-trajectory groups and mortality is concordant with our results. Phyo et al. classified four trajectory groups (high, intermediate, decline, low) over 6-year PCS in SF-12 using ASPREE trial participants with a mean age of 78.2 [40]. They also reported that declining PCS was associated with the risk of incident cardiovascular disease events and all-cause mortality [40]. Considering the general congruence among other previous studies which revealed that declining or low HRQOL is associated with poor health outcomes among various population [40–42], the association between the declining or low trajectory in the absolute score of PCS and LOI, compared with the high-stable group, is apparently reasonable. We believe that the current study is the first to identify out the association between HRQOL trajectories and the risk of LOI.

Although several previous studies have already revealed the predictive association between a declining trajectory of HRQOL and poor health outcomes, our study can be distinguished from previous studies by focusing on the change-score trajectory of HRQOL and revealing the association between an increasing trajectory in MCS change score and a lower risk of LOI among the population. Evidence regarding the trajectory of the change score of HRQOL among older adults is quite limited. We found only one study which described the HRQOL change score trajectory among older adults. Ejiri et al. investigated the trajectory of the change score of HRQOL among community-dwelling older adults using WHO-5 Well-Being Index during the two years of the COVID-19 pandemic [43]. Their GBTM also identified three trajectories and the shape of the trajectories of the change score of HRQOL is quite similar to our results. This similarity in the classification using the same analysis between two separate cohorts could support the validity of our results. Notably, the increase trajectory in MCS change score was independently associated with a lower risk of LOI, whereas the increase trajectory in PCS change score was not independently associated with a lower risk of LOI. Additionally, the decline trajectories in MCS and PCS were independently associated with higher risks of LOI, though the increase trajectory in the absolute MCS was not independently associated with a lower risk of LOI. These results might suggest that the HRQOL change score has greater sensitivity than its absolute score. In other words, the relative score within an individual can be a more sensitive predictor than its absolute score. Several previous studies have also reported that the change score in HRQOL is a sensitive indicator for predicting at least mortality among older adults [42, 44, 45].

Although the association between an increase trajectory in MCS change score and a lower risk of LOI cannot be interpreted as causality, our results could bridge the current evidence gap between improvement in HRQOL and better health outcomes among community-dwelling older adults. Previous studies have detected that various factors which significantly affect older adults’ HRQOL [46–49]. This means that diverse and non-uniform factors underlying the increase HRQOL trajectory could explain the association. The existence of non-uniformity in potential interventions across the participants could violate the consistency assumption and could skew the value of the association to some extent [50]. We have rigorously followed up with the participants since 2017 and adjusted for as many baseline confounders as we possible could, but we could not obtain individual episodes as time-varying variables, which can potentially affect the participants’ HRQOL and outcomes during the follow-up period. However, as an initial phase of research trying to forge a new theory, sometimes it may be appropriate to back-burner consistency [50]. We believe our results support the possibility that improvement in HRQOL could be associated with a lower risk of LOI in a real-world setting. An intraindividual improvement trajectory in HRQOL can be one possible natural course during the late-life stage. Moreover, an improvement trajectory, especially in MCS, could be associated with a lower risk of LOI. More studies are necessary to confirm this hypothesis.

#### Strengths and limitations of this study

Our study has several strengths. First, the population of this study (community-dwelling individuals aged ≥ 75) is distinguished from those in other previous studies. The trajectories of HRQOL among this population have never been delineated before this study. Second, we successfully maintained a certain level of response rate among the population during the observation period. Continuous HRQOL data acquisition was necessary to answer the clinical question in this study. Third, the outcome data in this study were authorized by the municipal government, which certifies the outcomes for participants. Long-term collaborative work with the municipal government, hospital, and the academic sector brought this study to fruition. This study also has several limitations. First, our study population was derived from only one municipality; thus, the generalizability of our results is not guaranteed. Second, although the trajectory was never observed after the outcome, the estimated association in the main analysis cannot be interpreted as not only causal but also a predictive association in this study, since trajectories and outcomes were simultaneously observed over the same period. However, the results of sensitivity analysis 1, conducted for only outcomes that occurred after 60 months, were in line with the results of the main analysis. Thus, we believe the association in sensitivity analysis 1 could be interpreted as a predictive association, whereas the main aim of this study was not to evaluate the predictiveness of the HRQOL but to evaluate the association between HRQOL trajectories and outcomes in a more comprehensive context. Third, as living humans inherently encompass both mental and physical components, we conducted a 9-class stratified analysis in an exploratory manner. However, due to insufficient sample sizes in certain classes, it was not possible to estimate robust associations, and some classes failed to converge. Fourth, our data did not include information regarding the reasons for LOI and death. Thus, we could not investigate the association further according to the reasons for those outcomes.

## Conclusion

In conclusion, the longitudinal trajectory of HRQOL is closely related to LOI among the community-dwelling older adults. The decline trajectory in the physical and mental components has a relatively strong association with higher risk of LOI; however, an intraindividual increase trajectory in the mental component could be associated with a lower risk of LOI. This study delineated the possibility of HRQOL improvement among older adults and underscored the importance of improving the mental component of HRQOL from the perspective of blessing the independence.

## Supplementary Information

Below is the link to the electronic supplementary material.

Supplementary File 1 (DOCX 3.95 MB)

## Data Availability

The datasets used and analyzed during the study are available from the corresponding author upon request and subject to ethical approval.

## Abbreviations

aHR: Adjusted Hazard ratio
BIC: Bayesian information criterion
CI: Confidence interval
GBTM: Group-based trajectory modeling
HLE: Healthy life expectancy
HR: Hazard ratio
HRQOL: Health-related quality of life
IQR: Interquartile range
LE: Life expectancy
LOI: Loss of independence
LTCI: Long-Term Care Insurance
MCS: Mental component scale
PCS: Physical component scale
SARC-F: Strength, assistance with walking, rising from a chair, climbing stairs, and falls. (A five-item questionnaire used as a screening tool to identify individuals at risk for sarcopenia)
SF-8: Short-Form Health Survey-8 [a short version of the original 36-item Short-Form Health Survey (SF-36)]
UCLA: University of California, Los Angeles

## Declarations

### Disclaimer

The funders had no role in study design, data collection and analysis, decision to publish, or preparation of the manuscript.
