# Supplementary material for: Health-related quality of life trajectories and loss of independence among community-dwelling older adults
Source: GeroScience. 2025 Jul 29;48(2):2757–72. doi: 10.1007/s11357-025-01804-5 (PMC12972409; doi:10.1007/s11357-025-01804-5)
Supplement: Supplementary file 1 — (DOCX 3.95 MB) [file 11357_2025_1804_MOESM1_ESM.docx]

**SUPPLEMENTARY MATERIALS**

# **Title: Quality of life trajectories and loss of independence among community living older people**

**CONTENTS**

**Supplementary Table 1.** Model diagnostics for group-based trajectory models of **MCS** in SF-8

**Supplementary Table 2.** Model diagnostics for group-based trajectory models of **PCS** in SF-8

**Supplementary Table 3.** Model diagnostics for group-based trajectory models of **MCS change score**

**Supplementary Table 4.** Model diagnostics for group-based trajectory models of **PCS change score**

**Supplementary Figure 1.** Individual observed **MCS** trajectories of randomly selected participants in each class

**Supplementary Figure 2.** Individual observed **PCS** trajectories of randomly selected participants in each class

**Supplementary Figure 3.** Individual observed trajectories **MCS change score** of randomly selected participants in each class

**Supplementary Figure 4.** Individual observed trajectories **PCS change score** of randomly selected participants in each class

**Supplementary Table 5**. Baseline characteristics of the eligible and ineligible patients

**Supplementary Table 6**. Outcome counts and incidence rates of the eligible and ineligible patients

**Supplementary Table 7**. Baseline characteristics of the patients stratified by classification of **MCS** trajectories

**Supplementary Table 8.** Baseline characteristics of the patients stratified by classification of **PCS** trajectories

**Supplementary Table 9**. Baseline characteristics of the patients stratified by classification of **MCS change score** trajectories

**Supplementary Table 10**. Baseline characteristics of the patients stratified by classification of **PCS change score** trajectories

**Supplementary Figure 5**. Kaplan-Meier survival estimates for composite outcome–free survival in each MCS trajectory

**Supplementary Figure 6**. Kaplan-Meier survival estimates for composite outcome–free survival in each PCS trajectory

**Supplementary Figure 7**. Kaplan-Meier survival estimates for composite outcome–free survival in each MCS change score trajectory

**Supplementary Figure 8**. Kaplan-Meier survival estimates for composite outcome–free survival in each PCS change score trajectory

**Supplementary Figure 9**. Kaplan-Meier survival estimates for composite outcome–free survival stratified by MCS and PCS trajectories

**Supplementary Figure 10**. Kaplan-Meier survival estimates for composite outcome–free survival stratified by MCS and PCS change score trajectories

**Supplementary Table 11.** Association of outcomes occurring after 60 months with trajectory patterns of HRQOL (Sensitivity analysis 1)

**Supplementary Table 12**. Association of outcomes with trajectory patterns of HRQOL among participants who were independent at baseline (Sensitivity analysis 2)

**Supplementary Table 1.** Model diagnostics for group-based trajectory models of **MCS** in SF-8

| Class | BIC | Class percentages (%) | | | | | | | Odds of correct classification for each trajectory | | | | | | | Average posterior probability per class  (higher is better) | | | | | | | Model average posterior probability for assigned class  (higher is better) | Relative Entropy  (close to 1 is better) |
| --- | --- | --- | --- | --- | --- | --- | --- | --- | --- | --- | --- | --- | --- | --- | --- | --- | --- | --- | --- | --- | --- | --- | --- | --- |
| Linear |  | 1 | 2 | 3 | 4 | 5 | 6 | 7 | 1 | 2 | 3 | 4 | 5 | 6 | 7 | 1 | 2 | 3 | 4 | 5 | 6 | 7 |  |  |
| 1 | 121059.4 | 100 |  |  |  |  |  |  | NA |  |  |  |  |  |  | 1.00 |  |  |  |  |  |  | 1.00 | 1.00 |
| 2 | 120524.4 | 6.1 | 93.9 |  |  |  |  |  | 40.1 | 2.2 |  |  |  |  |  | 0.79 | 0.96 |  |  |  |  |  | 0.88 | 0.81 |
| 3 | 119931.5 | 8.3 | 80.0 | 11.7 |  |  |  |  | 25.9 | 3.7 | 23.8 |  |  |  |  | 0.76 | 0.92 | 0.77 |  |  |  |  | 0.82 | 0.75 |
| 4 | 120575.8 | 7.6 | 92.4 | 0 | 0 |  |  |  | 26.8 | 1.1 | NA | NA |  |  |  | 0.72 | 0.56 | NA | NA |  |  |  | NA | 0.26 |
| 5 | 119982.8 | 11.2 | 0 | 74.4 | 14.3 | 0 |  |  | 15.6 | NA | 1.4 | 16.2 | NA |  |  | 0.66 | NA | 0.44 | 0.70 | NA |  |  | NA | 0.33 |
| 6 | 119777.3 | 5.4 | 3.2 | 0 | 27.3 | 64.0 | 0 |  | 37.4 | 132.0 | NA | 8.4 | 1.8 | NA |  | 0.73 | 0.83 | NA | 0.72 | 0.47 | NA |  | NA | 0.44 |
| 7 | 120034.2 | 13.1 | 15.7 | 71.3 | 0 | 0 | 0 | 0 | 12.3 | 13.7 | 1.4 | NA | NA | NA | NA | 0.60 | 0.66 | 0.29 | NA | NA | NA | NA | NA | 0.24 |
| Quadratic |  | 1 | 2 | 3 | 4 | 5 | 6 | 7 | 1 | 2 | 3 | 4 | 5 | 6 | 7 | 1 | 2 | 3 | 4 | 5 | 6 | 7 |  |  |
| 1 | 121067.8 | 100 |  |  |  |  |  |  | NA |  |  |  |  |  |  | 1.00 |  |  |  |  |  |  | 1.00 | 1.00 |
| 2 | 121084.9 | 43.3 | 56.7 |  |  |  |  |  | 1.0 | 1.0 |  |  |  |  |  | 0.50 | 0.50 |  |  |  |  |  | 0.50 | 0.0 |
| 3 | 119915.7 | 10.0 | 78.7 | 11.2 |  |  |  |  | 27.1 | 3.9 | 19.0 |  |  |  |  | 0.77 | 0.93 | 0.75 |  |  |  |  | 0.81 | 0.75 |
| 4 | 121119.2 | 42.7 | 0.4 | 0.7 | 56.2 |  |  |  | 1.0 | 1.0 | 1.0 | 1.0 |  |  |  | 0.26 | 0.25 | 0.25 | 0.26 |  |  |  | 0.25 | 0.0 |
| 5 | 121136.3 | 42.4 | 0.4 | 0.7 | 0.5 | 56.0 |  |  | 1.0 | 1.0 | 1.0 | 1.0 | 1.0 |  |  | 0.21 | 0.20 | 0.20 | 0.20 | 0.21 |  |  | 0.20 | 0.0 |
| 6 | 119724.7 | 11.7 | 10.6 | 4.7 | 73.0 | 0 | 0 |  | 14.2 | 21.3 | 32.7 | 1.5 | NA | NA |  | 0.62 | 0.70 | 0.67 | 0.42 | NA |  |  | NA | 0.39 |
| 7 | 119761.4 | 10.3 | 13.3 | 5.3 | 0 | 0 | 71 | 0 | 15.3 | 15.7 | 26.2 | NA | NA | 1.4 | NA | 0.60 | 0.66 | 0.62 | NA | NA | 0.31 | NA | NA | 0.31 |
| Cubic |  | 1 | 2 | 3 | 4 | 5 | 6 | 7 | 1 | 2 | 3 | 4 | 5 | 6 | 7 | 1 | 2 | 3 | 4 | 5 | 6 | 7 |  |  |
| 1 | 121071.1 | 100 |  |  |  |  |  |  | NA |  |  |  |  |  |  | 1.00 |  |  |  |  |  |  | 1.00 | 1.00 |
| 2 | 120501.4 | 6.2 | 93.8 |  |  |  |  |  | 43.2 | 2.3 |  |  |  |  |  | 0.81 | 0.96 |  |  |  |  |  | 0.89 | 0.81 |
| 3* | 119902.8 | 11.9 | 79.3 | 8.8 |  |  |  |  | 26.5 | 4.7 | 30.8 |  |  |  |  | 0.77 | 0.93 | 0.77 |  |  |  |  | 0.82 | 0.76 |
| 4 | 119617.9 | 8.5 | 78.4 | 8.2 | 5.0 |  |  |  | 22.8 | 3.7 | 31.8 | 45.3 |  |  |  | 0.72 | 0.92 | 0.76 | 0.77 |  |  |  | 0.79 | 0.77 |
| 5 | 119660.6 | 8.4 | 9.2 | 5.3 | 77.0 | 0 |  |  | 29.8 | 20.3 | 40.5 | 2.0 | NA |  |  | 0.74 | 0.70 | 0.75 | 0.75 | NA |  |  | NA | 0.58 |
| 6 | 119703.4 | 9.0 | 10.6 | 0 | 5.9 | 74.5 | 0 |  | 26.3 | 16.5 | NA | 32.6 | 1.5 | NA |  | 0.72 | 0.65 | NA | 0.71 | 0.52 | NA |  | NA | 0.41 |
| 7 | 119746.2 | 9.6 | 11.8 | 0 | 0 | 6.5 | 72.1 | 0 | 23.1 | 13.9 | NA | NA | 27.3 | 1.4 | NA | 0.69 | 0.61 | NA | NA | 0.67 | 0.37 | NA | NA | 0.76 |

An asterisk (*) indicates the final model.

**Supplementary Table 2.** Model diagnostics for group-based trajectory models of **PCS** in SF-8

| Class | BIC | Class percentages (%) | | | | | | | Odds of correct classification for each trajectory | | | | | | | Average posterior probability per class  (higher is better) | | | | | | | Model average posterior probability for assigned class  (higher is better) | Relative Entropy  (close to 1 is better) |
| --- | --- | --- | --- | --- | --- | --- | --- | --- | --- | --- | --- | --- | --- | --- | --- | --- | --- | --- | --- | --- | --- | --- | --- | --- |
| Linear |  | 1 | 2 | 3 | 4 | 5 | 6 | 7 | 1 | 2 | 3 | 4 | 5 | 6 | 7 | 1 | 2 | 3 | 4 | 5 | 6 | 7 |  |  |
| 1 | 123956.0 | 100 |  |  |  |  |  |  | NA |  |  |  |  |  |  | 1.00 |  |  |  |  |  |  | 1.00 | 1.00 |
| 2 | 123328.1 | 18.0 | 82.0 |  |  |  |  |  | 21.3 | 4.3 |  |  |  |  |  | 0.84 | 0.95 |  |  |  |  |  | 0.90 | 0.74 |
| 3 | 122955.0 | 12.3 | 80.5 | 7.1 |  |  |  |  | 24.1 | 4.0 | 28.9 |  |  |  |  | 0.79 | 0.93 | 0.75 |  |  |  |  | 0.82 | 0.77 |
| 4 | 123379.4 | 21.7 | 0 | 78.3 | 0 |  |  |  | 13.4 | NA | 1.4 | NA |  |  |  | 0.77 | NA | 0.47 | NA |  |  |  | NA | 0.26 |
| 5 | 123405.1 | 23.7 | 76.3 | 0 | 0 | 0 |  |  | 10.9 | 1.3 | NA | NA | NA |  |  | 0.72 | 0.34 | NA | NA | NA |  |  | NA | 0.21 |
| 6 | 123032.0 | 14.4 | 0 | 8.3 | 0 | 77.3 | 0 |  | 17.4 | NA | 22.5 | NA | 1.4 | NA |  | 0.73 | NA | 0.69 | NA | 0.50 | NA |  | NA | 0.34 |
| 7 | 123057.7 | 16.3 | 0 | 9.9 | 0 | 0 | 73.8 | 0 | 13.5 | NA | 16.7 | NA | NA | 1.3 | NA | 0.68 | NA | 0.63 | NA | NA | 0.29 | NA | NA | 0.25 |
| Quadratic |  | 1 | 2 | 3 | 4 | 5 | 6 | 7 | 1 | 2 | 3 | 4 | 5 | 6 | 7 | 1 | 2 | 3 | 4 | 5 | 6 | 7 |  |  |
| 1 | 123853.0 | 100 |  |  |  |  |  |  | NA |  |  |  |  |  |  | 1.00 |  |  |  |  |  |  | 1.00 | 1.00 |
| 2 | 123222.8 | 17.7 | 82.3 |  |  |  |  |  | 22.0 | 4.0 |  |  |  |  |  | 0.84 | 0.94 |  |  |  |  |  | 0.89 | 0.74 |
| 3 | 122806.8 | 8.6 | 80.5 | 11.0 |  |  |  |  | 33.6 | 4.2 | 22.5 |  |  |  |  | 0.78 | 0.94 | 0.76 |  |  |  |  | 0.83 | 0.77 |
| 4 | 122557.8 | 8.9 | 4.1 | 79.2 | 7.7 |  |  |  | 32.0 | 40.1 | 3.9 | 27.5 |  |  |  | 0.78 | 0.72 | 0.92 | 0.73 |  |  |  | 0.79 | 0.77 |
| 5 | 122402.3 | 9.5 | 1.8 | 79.1 | 4.3 | 5.4 |  |  | 26.2 | 124.1 | 4.0 | 39.9 | 35.7 |  |  | 0.74 | 0.75 | 0.92 | 0.73 | 0.71 |  |  | 0.77 | 0.77 |
| 6 | 122436.5 | 1.9 | 9.6 | 4.4 | 78.7 | 5.4 | 0 |  | 118.3 | 25.6 | 38.6 | 2.3 | 35.0 | NA |  | 0.74 | 0.74 | 0.72 | 0.82 | 0.71 | NA |  | NA | 0.67 |
| 7 | 122660.5 | 10.5 | 5.1 | 0 | 9.5 | 0 | 74.9 | 0 | 22.7 | 28.2 | NA | 19.4 | NA | 1.4 | NA | 0.71 | 0.64 | NA | 0.66 | NA | 0.41 | NA | NA | 0.34 |
| Cubic |  | 1 | 2 | 3 | 4 | 5 | 6 | 7 | 1 | 2 | 3 | 4 | 5 | 6 | 7 | 1 | 2 | 3 | 4 | 5 | 6 | 7 |  |  |
| 1 | 123847.8 | 100 |  |  |  |  |  |  | NA |  |  |  |  |  |  | 1.00 |  |  |  |  |  |  | 1.00 | 1.00 |
| 2 | 123229.6 | 17.9 | 82.1 |  |  |  |  |  | 21.2 | 4.0 |  |  |  |  |  | 0.84 | 0.94 |  |  |  |  |  | 0.89 | 0.73 |
| 3* | 122815.1 | 11.4 | 8.7 | 80.0 |  |  |  |  | 26.3 | 4.4 | 35.4 |  |  |  |  | 0.78 | 0.77 | 0.93 |  |  |  |  | 0.83 | 0.77 |
| 4 | 122524.4 | 8.6 | 7.7 | 78.7 | 5.0 |  |  |  | 33.4 | 26.8 | 4.1 | 42.2 |  |  |  | 0.78 | 0.72 | 0.93 | 0.76 |  |  |  | 0.81 | 0.78 |
| 5 | 122422.3 | 9.2 | 1.6 | 78.2 | 4.9 | 6.1 |  |  | 29.1 | 96.2 | 3.9 | 31.8 | 31.89 |  |  | 0.76 | 0.72 | 0.92 | 0.70 | 0.71 |  |  | 0.76 | 0.78 |
| 6 | 122347.6 | 7.7 | 1.0 | 2.5 | 77.8 | 3.1 | 8.0 |  | 35.3 | 178.6 | 55.3 | 4.0 | 54.1 | 21.1 |  | 0.76 | 0.73 | 0.67 | 0.92 | 0.74 | 0.66 |  | 0.75 | 0.79 |
| 7 | 122161.3 | 8.2 | 0.8 | 2.1 | 3.6 | 2.8 | 77.4 | 5.2 | 29.9 | 212.3 | 127.5 | 46.7 | 56.3 | 3.9 | 30.4 | 0.73 | 0.70 | 0.79 | 0.73 | 0.69 | 0.91 | 0.66 | 0.74 | 0.80 |

An asterisk (*) indicates the final model.

**Supplementary Table 3.** Model diagnostics for group-based trajectory models of **MCS change score**

| Class | BIC | Class percentages (%) | | | | | | | Odds of correct classification for each trajectory | | | | | | | Average posterior probability per class  (higher is better) | | | | | | | Model average posterior probability for assigned class  (higher is better) | Relative Entropy  (close to 1 is better) |
| --- | --- | --- | --- | --- | --- | --- | --- | --- | --- | --- | --- | --- | --- | --- | --- | --- | --- | --- | --- | --- | --- | --- | --- | --- |
| Linear |  | 1 | 2 | 3 | 4 | 5 | 6 | 7 | 1 | 2 | 3 | 4 | 5 | 6 | 7 | 1 | 2 | 3 | 4 | 5 | 6 | 7 |  |  |
| 1 | 111563.7 | 100 |  |  |  |  |  |  | NA |  |  |  |  |  |  | 1.00 |  |  |  |  |  |  | 1.00 | 1.00 |
| 2 | 110347.6 | 11.5 | 88.5 |  |  |  |  |  | 27.2 | 3.0 |  |  |  |  |  | 0.82 | 0.95 |  |  |  |  |  | 0.89 | 0.76 |
| 3 | 109203.5 | 10.8 | 82.9 | 6.3 |  |  |  |  | 40.2 | 3.9 | 80.8 |  |  |  |  | 0.85 | 0.94 | 0.86 |  |  |  |  | 0.88 | 0.83 |
| 4 | 108929.7 | 3.8 | 73.5 | 16.6 | 6.1 |  |  |  | 114.1 | 3.6 | 12.4 | 96.9 |  |  |  | 0.84 | 0.89 | 0.75 | 0.87 |  |  |  | 0.84 | 0.76 |
| 5 | 108734.7 | 4.1 | 16.0 | 7.3 | 70.4 | 2.2 |  |  | 116.7 | 14.7 | 37.9 | 3.7 | 289.3 |  |  | 0.85 | 0.77 | 0.78 | 0.88 | 0.87 |  |  | 0.83 | 0.77 |
| 6 | 108715.8 | 4.1 | 16.1 | 70.2 | 2.1 | 7.4 | 0.2 |  | 118.1 | 14.7 | 3.7 | 242.9 | 35.3 | 4350.5 |  | 0.85 | 0.77 | 0.88 | 0.85 | 0.77 | 0.90 |  | 0.84 | 0.79 |
| 7 | 108709.8 | 0.8 | 4.6 | 63.4 | 0 | 20.4 | 8.6 | 2.2 | 391.1 | 57.4 | 1.8 | NA | 8.5 | 28.5 | 288.0 | 0.80 | 0.76 | 0.48 | NA | 0.67 | 0.72 | 0.87 | NA | 0.56 |
| Quadratic |  | 1 | 2 | 3 | 4 | 5 | 6 | 7 | 1 | 2 | 3 | 4 | 5 | 6 | 7 | 1 | 2 | 3 | 4 | 5 | 6 | 7 |  |  |
| 1 | 111571.9 | 100 |  |  |  |  |  |  | NA |  |  |  |  |  |  | 1.00 |  |  |  |  |  |  | 1.00 | 1.00 |
| 2 | 110041.3 | 13.6 | 86.4 |  |  |  |  |  | 28.9 | 3.6 |  |  |  |  |  | 0.84 | 0.95 |  |  |  |  |  | 0.90 | 0.77 |
| 3* | 108298.7 | 12.3 | 80.0 | 7.6 |  |  |  |  | 44.4 | 5.2 | 89.0 |  |  |  |  | 0.87 | 0.95 | 0.89 |  |  |  |  | 0.90 | 0.85 |
| 4 | 107817.9 | 7.8 | 75.4 | 9.3 | 7.5 |  |  |  | 52.2 | 4.2 | 20.3 | 100.9 |  |  |  | 0.82 | 0.92 | 0.74 | 0.90 |  |  |  | 0.85 | 0.80 |
| 5 | 107541.4 | 2.5 | 8.2 | 72.2 | 9.5 | 7.5 |  |  | 205.9 | 22.8 | 4.3 | 23.4 | 109.7 |  |  | 0.86 | 0.74 | 0.90 | 0.73 | 0.91 |  |  | 0.83 | 0.79 |
| 6 | 107401.1 | 3.0 | 66.2 | 15.8 | 5.7 | 1.9 | 7.4 |  | 139.8 | 4.0 | 9.1 | 34.3 | 114.4 | 111.2 |  | 0.83 | 0.86 | 0.68 | 0.70 | 0.77 | 0.90 |  | 0.79 | 0.75 |
| 7 | 106987.5 | 2.2 | 10.1 | 9.1 | 65.3 | 9.0 | 1.6 | 2.7 | 248.7 | 15.1 | 26.5 | 4.1 | 39.7 | 154.3 | 254.7 | 0.87 | 0.70 | 0.73 | 0.86 | 0.82 | 0.77 | 0.89 | 0.81 | 0.77 |
| Cubic |  | 1 | 2 | 3 | 4 | 5 | 6 | 7 | 1 | 2 | 3 | 4 | 5 | 6 | 7 | 1 | 2 | 3 | 4 | 5 | 6 | 7 |  |  |
| 1 | 111575.5 | 100 |  |  |  |  |  |  | NA |  |  |  |  |  |  | 1.00 |  |  |  |  |  |  | 1.00 | 1.00 |
| 2 | 110038.0 | 15.0 | 85.0 |  |  |  |  |  | 25.4 | 3.7 |  |  |  |  |  | 0.84 | 0.95 |  |  |  |  |  | 0.90 | 0.73 |
| 3 | 108073.4 | 12.8 | 79.2 | 7.9 |  |  |  |  | 43.6 | 5.5 | 88.6 |  |  |  |  | 0.87 | 0.95 | 0.89 |  |  |  |  | 0.90 | 0.77 |
| 4 | 107536.7 | 8.6 | 76.6 | 6.8 | 8.0 |  |  |  | 48.8 | 4.8 | 38.0 | 92.6 |  |  |  | 0.83 | 0.93 | 0.78 | 0.90 |  |  |  | 0.81 | 0.78 |
| 5 | 107108.9 | 2.8 | 71.3 | 8.1 | 9.9 | 8.0 |  |  | 186.1 | 4.8 | 34.1 | 25.1 | 98.9 |  |  | 0.86 | 0.91 | 0.76 | 0.78 | 0.90 |  |  | 0.86 | 0.78 |
| 6 | 106653.3 | 2.7 | 10.2 | 66.8 | 8.0 | 9.3 | 2.9 |  | 223.0 | 33.0 | 4.6 | 37.9 | 30.0 | 291.7 |  | 0.88 | 0.81 | 0.89 | 0.78 | 0.79 | 0.90 |  | 0.84 | 0.79 |
| 7 | 106382.7 | 1.5 | 9.8 | 64.1 | 12.3 | 5.9 | 3.6 | 2.8 | 396.6 | 35.2 | 4.8 | 17.7 | 39.1 | 126.7 | 345.2 | 0.87 | 0.81 | 0.88 | 0.73 | 0.75 | 0.84 | 0.92 | 0.83 | 0.80 |

An asterisk (*) indicates the final model.

**Supplementary Table 4.** Model diagnostics for group-based trajectory models of **PCS change score**

| Class | BIC | Class percentages (%) | | | | | | | Odds of correct classification for each trajectory | | | | | | | Average posterior probability per class  (higher is better) | | | | | | | Model average posterior probability for assigned class  (higher is better) | Relative Entropy  (close to 1 is better) |
| --- | --- | --- | --- | --- | --- | --- | --- | --- | --- | --- | --- | --- | --- | --- | --- | --- | --- | --- | --- | --- | --- | --- | --- | --- |
| Linear |  | 1 | 2 | 3 | 4 | 5 | 6 | 7 | 1 | 2 | 3 | 4 | 5 | 6 | 7 | 1 | 2 | 3 | 4 | 5 | 6 | 7 |  |  |
| 1 | 112209.4 | 100 |  |  |  |  |  |  | NA |  |  |  |  |  |  | 1.00 |  |  |  |  |  |  | 1.00 | 1.00 |
| 2 | 110967.4 | 13.6 | 86.4 |  |  |  |  |  | 22.5 | 2.9 |  |  |  |  |  | 0.82 | 0.94 |  |  |  |  |  | 0.88 | 0.72 |
| 3 | 110228.5 | 11.6 | 83.5 | 4.9 |  |  |  |  | 34.5 | 3.3 | 84.8 |  |  |  |  | 0.85 | 0.93 | 0.84 |  |  |  |  | 0.87 | 0.81 |
| 4 | 109886.1 | 3.0 | 20.6 | 4.6 | 71.7 |  |  |  | 150.8 | 10.9 | 107.8 | 3.4 |  |  |  | 0.84 | 0.77 | 0.86 | 0.88 |  |  |  | 0.84 | 0.75 |
| 5 | 109704.7 | 3.5 | 19.1 | 68.8 | 7.5 | 1.1 |  |  | 143.7 | 13.1 | 3.5 | 37.0 | 532.8 |  |  | 0.85 | 0.79 | 0.86 | 0.79 | 0.87 |  |  | 0.83 | 0.76 |
| 6 | 109596.8 | 1.0 | 4.3 | 7.5 | 21.2 | 65.0 | 1.1 |  | 426.1 | 69.3 | 40.8 | 9.9 | 3.5 | 571.1 |  | 0.82 | 0.79 | 0.80 | 0.76 | 0.84 | 0.88 |  | 0.82 | 0.76 |
| 7 | 109523.5 | 1.0 | 4.2 | 20.7 | 64.9 | 7.8 | 1.4 | 0.04 | 426.8 | 71.0 | 10.2 | 3.5 | 33.8 | 402.5 | 15639853 | 0.82 | 0.79 | 0.76 | 0.84 | 0.78 | 0.87 | 1 | 0.84 | 0.77 |
| Quadratic |  | 1 | 2 | 3 | 4 | 5 | 6 | 7 | 1 | 2 | 3 | 4 | 5 | 6 | 7 | 1 | 2 | 3 | 4 | 5 | 6 | 7 |  |  |
| 1 | 112101.2 | 100 |  |  |  |  |  |  | NA |  |  |  |  |  |  | 1.00 |  |  |  |  |  |  | 1.00 | 1.00 |
| 2 | 110407.2 | 16.0 | 84.0 |  |  |  |  |  | 23.7 | 3.5 |  |  |  |  |  | 0.84 | 0.94 |  |  |  |  |  | 0.89 | 0.74 |
| 3* | 109126.4 | 13.0 | 80.9 | 6.1 |  |  |  |  | 40.5 | 4.2 | 95.3 |  |  |  |  | 0.87 | 0.94 | 0.88 |  |  |  |  | 0.90 | 0.83 |
| 4 | 108581.8 | 4.6 | 65.4 | 24.8 | 5.1 |  |  |  | 121.2 | 4.3 | 10.5 | 134.0 |  |  |  | 0.86 | 0.88 | 0.80 | 0.89 |  |  |  | 0.86 | 0.76 |
| 5 | 108303.3 | 3.3 | 16.5 | 5.6 | 69.2 | 5.4 |  |  | 189.0 | 12.0 | 31.4 | 4.1 | 139.0 |  |  | 0.87 | 0.72 | 0.74 | 0.88 | 0.90 |  |  | 0.82 | 0.76 |
| 6 | 107882.1 | 3.2 | 65.8 | 13.7 | 5.7 | 9.8 | 1.7 |  | 209.8 | 4.1 | 17.3 | 33.2 | 36.9 | 490.3 |  | 0.88 | 0.86 | 0.75 | 0.74 | 0.83 | 0.90 |  | 0.83 | 0.77 |
| 7 | 107709.3 | 1.8 | 10.0 | 12.0 | 9.3 | 62.9 | 2.3 | 1.7 | 296.0 | 14.7 | 18.2 | 42.1 | 4.0 | 120.9 | 517.4 | 0.85 | 0.71 | 0.72 | 0.84 | 0.84 | 0.78 | 0.90 | 0.81 | 0.75 |
| Cubic |  | 1 | 2 | 3 | 4 | 5 | 6 | 7 | 1 | 2 | 3 | 4 | 5 | 6 | 7 | 1 | 2 | 3 | 4 | 5 | 6 | 7 |  |  |
| 1 | 112097.9 | 100 |  |  |  |  |  |  | NA |  |  |  |  |  |  | 1.00 |  |  |  |  |  |  | 1.00 | 1.00 |
| 2 | 110352.0 | 18.3 | 81.7 |  |  |  |  |  | 20.2 | 3.6 |  |  |  |  |  | 0.84 | 0.93 |  |  |  |  |  | 0.89 | 0.71 |
| 3 | 108875.8 | 14.0 | 79.2 | 6.9 |  |  |  |  | 37.1 | 4.5 | 87.7 |  |  |  |  | 0.87 | 0.94 | 0.88 |  |  |  |  | 0.90 | 0.83 |
| 4 | 108256.0 | 4.4 | 25.9 | 63.6 | 6.1 |  |  |  | 159.0 | 11.0 | 4.6 | 115.6 |  |  |  | 0.89 | 0.81 | 0.88 | 0.89 |  |  |  | 0.87 | 0.76 |
| 5 | 107905.5 | 3.8 | 20.3 | 65.2 | 4.5 | 6.2 |  |  | 166.9 | 11.2 | 4.4 | 52.0 | 119.1 |  |  | 0.88 | 0.77 | 0.87 | 0.77 | 0.90 |  |  | 0.84 | 0.77 |
| 6 | 107442.7 | 3.5 | 10.6 | 63.3 | 15.3 | 5.4 | 1.9 |  | 191.3 | 34.5 | 4.4 | 17.2 | 43.3 | 426.4 |  | 0.88 | 0.83 | 0.86 | 0.77 | 0.76 | 0.89 |  | 0.83 | 0.78 |
| 7 | 107231.3 | 2.1 | 51.6 | 7.1 | 25.3 | 8.4 | 4.0 | 1.5 | 304.0 | 4.4 | 39.5 | 6.6 | 46.0 | 77.5 | 589.5 | 0.88 | 0.80 | 0.77 | 0.71 | 0.83 | 0.79 | 0.91 | 0.81 | 0.73 |

An asterisk (*) indicates the final model.

**Supplementary Figure 1.** Individual observed **MCS** trajectories of randomly selected participants in each class

**
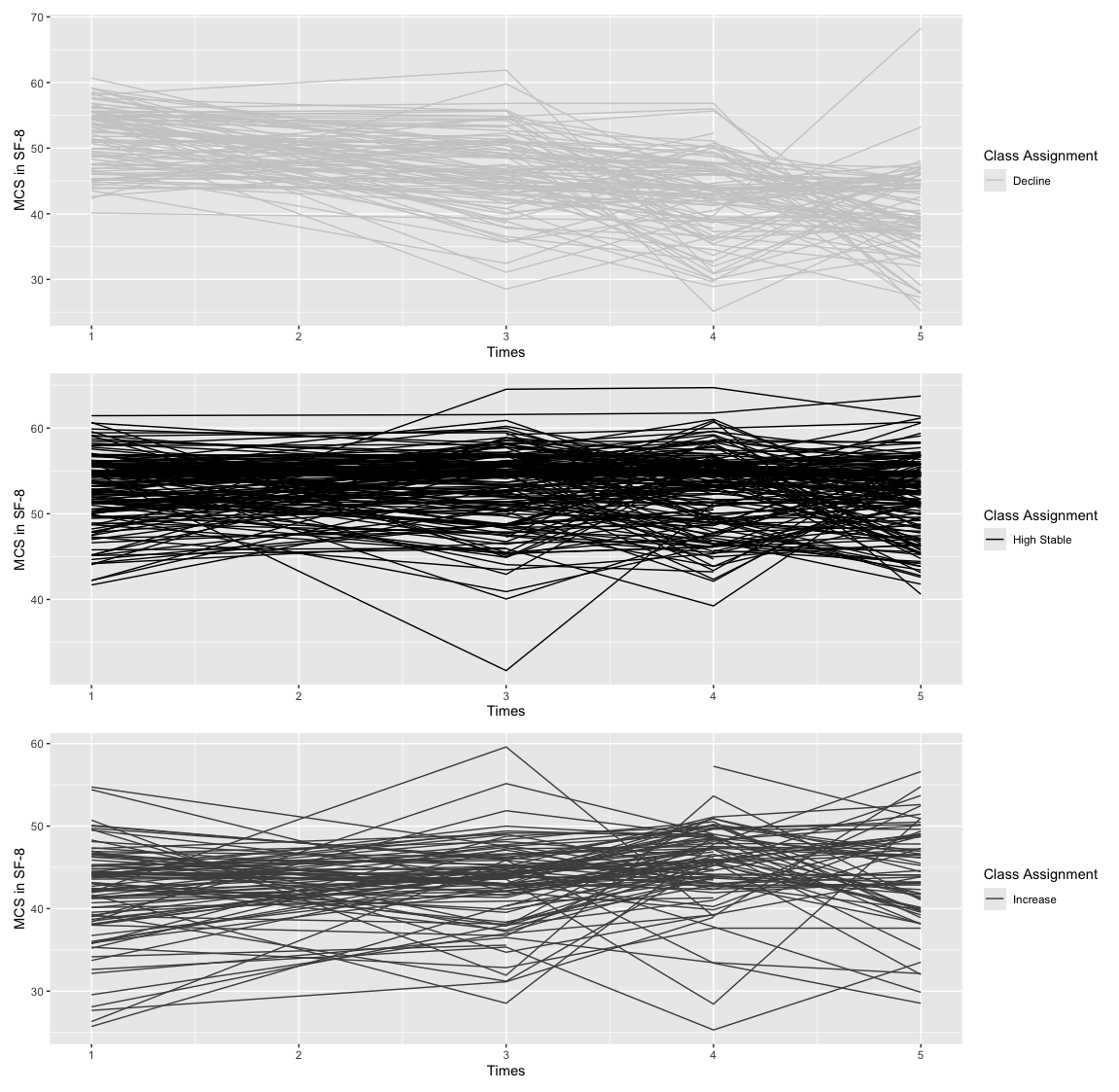
**

**Supplementary Figure 2.** Individual observed **PCS** trajectories of randomly selected participants in each class

**
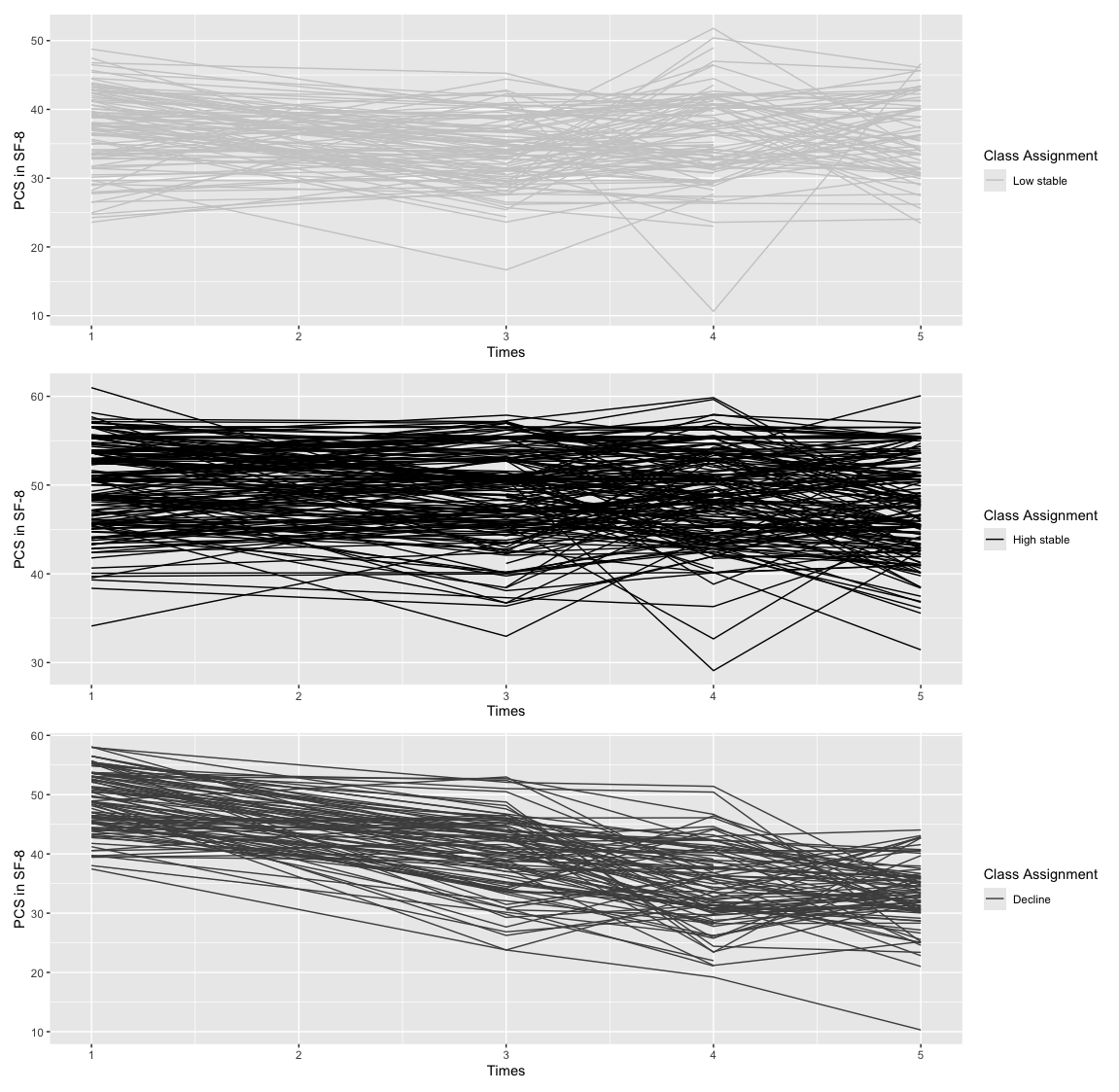
**

**Supplementary Figure 3.** Individual observed trajectories **MCS change score** of randomly selected participants in each class

**
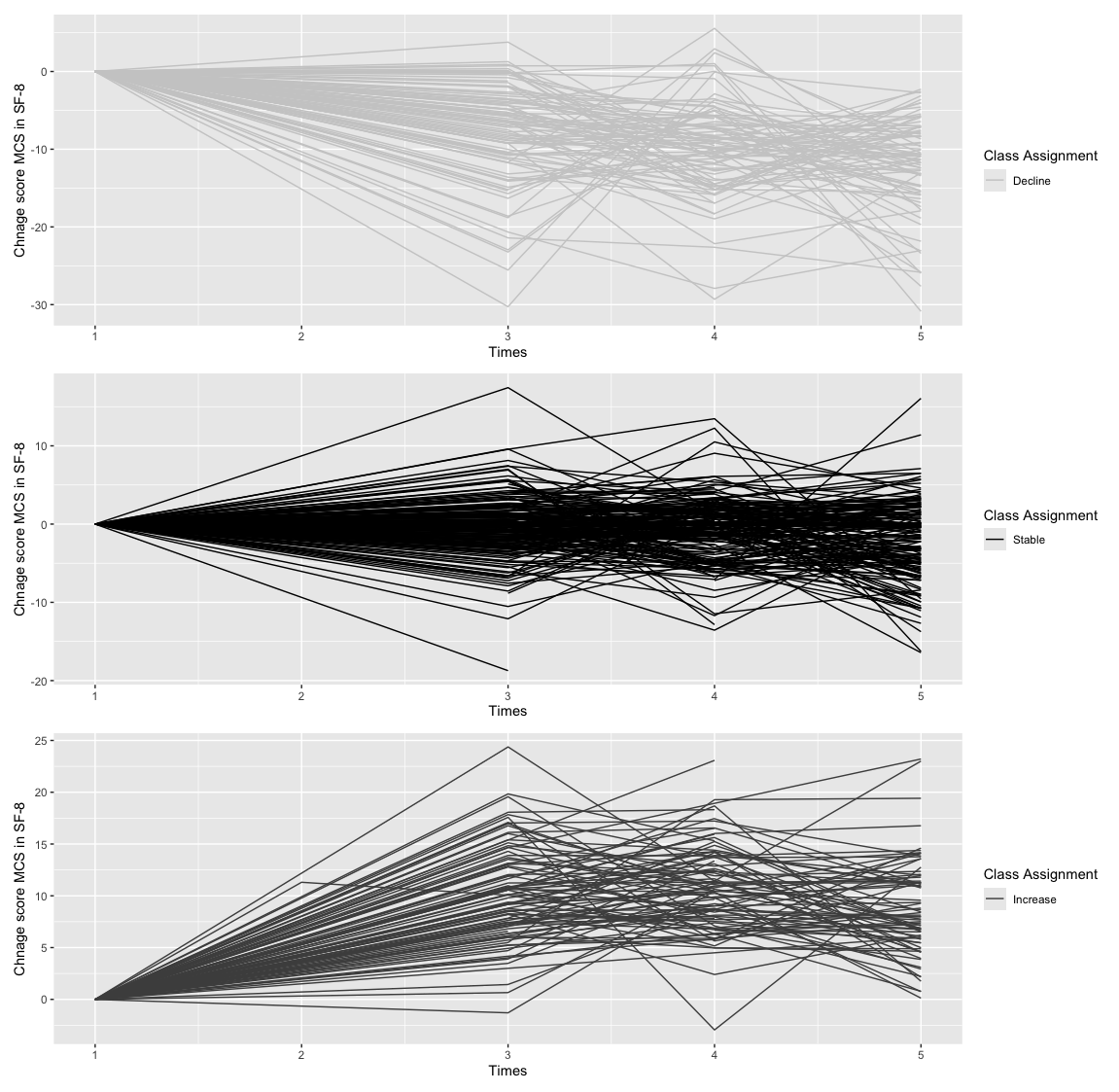
**

**Supplementary Figure 4.** Individual observed trajectories **PCS change score** of randomly selected participants in each class

**
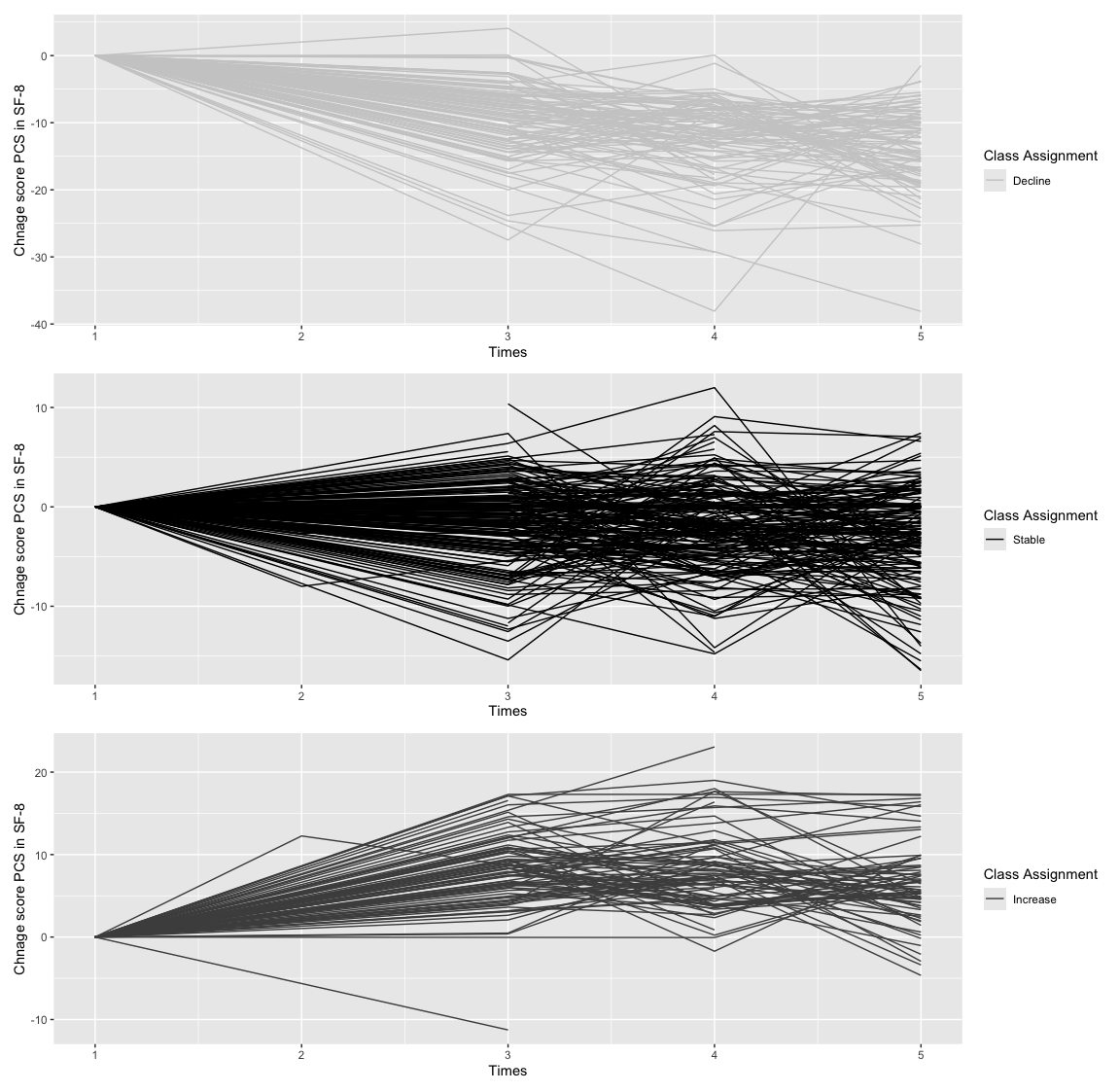
**

**Supplementary Table 5**. Baseline characteristics of the eligible and ineligible patients

| **Characteristics** | **Eligibles N = 4,043** | **Ineligibles N = 2,085** |
| --- | --- | --- |
| Age, (years), median (IQR) | 78.8 (75.8, 82.5) | 78.1 (72.6, 84.6) |
| Sex, n(%) |  |  |
| Men | 1,789 (44%) | 928 (45%) |
| Women | 2,254 (56%) | 1,157 (55%) |
| BMI, (kg/m^2), median (IQR) | 22.9 (20.8, 25.1) | 22.6 (20.5, 24.8) |
| Smoking habit, n(%) |  |  |
| Never | 2,499 (63%) | 1,203 (58%) |
| Past | 1,261 (32%) | 686 (33%) |
| Current | 220 (5.5%) | 169 (8.2%) |
| Missing | 63 | 27 |
| Alcohol habit, n(%) |  |  |
| Never | 2,877 (72%) | 1,511 (73%) |
| 1-2 days/week | 254 (6.3%) | 127 (6.2%) |
| 3-4 days/week | 226 (5.6%) | 93 (4.5%) |
| 5-6 days/week | 155 (3.9%) | 84 (4.1%) |
| Everyday | 497 (12%) | 248 (12%) |
| Missing | 34 | 22 |
| Unmarried, n(%) | 31 (0.8%) | 18 (0.9%) |
| Living alone, n(%) | 637 (16%) | 316 (15%) |
| Missing | 92 | 39 |
| Highest level of education, n(%) |  |  |
| Elementary school | 180 (4.9%) | 52 (4.6%) |
| Junior high school | 1,680 (46%) | 458 (40%) |
| High school | 1,199 (33%) | 428 (38%) |
| Junior college | 85 (2.3%) | 28 (2.5%) |
| Vocational school | 252 (6.8%) | 71 (6.3%) |
| University | 230 (6.2%) | 89 (7.8%) |
| Graduate school | 5 (0.1%) | 0 (0%) |
| Others | 50 (1.4%) | 10 (0.9%) |
| Missing | 362 | 949 |
| Annual household income < ￥3M ($20.6K), n(%) | 978 (24%) | 301 (14%) |
| UCLA loneliness scale ≥ 6, n(%) | 607 (16%) | 224 (20%) |
| Missing | 322 | 938 |
| SARC-F ≥ 4, n(%) | 1,021 (28%) | 229 (20%) |
| Missing | 384 | 941 |
| History of malignant disease, n(%) | 445 (11%) | 861 (41%) |
| History of myocardial infarction, n(%) | 536 (13%) | 911 (44%) |
| History of stroke, n(%) | 584 (14%) | 989 (47%) |
| History of depression, n(%) | 591 (15%) | 1,014 (49%) |
| Diabetes, n(%) | 499 (12%) | 877 (42%) |
| Baseline MCS score, median (IQR) | 52.9 (48.4, 55.0) | 51.3 (45.7, 54.8) |
| Baseline PCS score, median (IQR) | 50.0 (44, 54) | 48 (42, 53) |
| Baseline LTCI level, n(%) |  |  |
| Independent | 3,777 (93%) | 1,893 (91%) |
| Support care level 1 | 105 (2.6%) | 63 (3.0%) |
| Support care level 2 | 161 (4.0%) | 129 (6.2%) |
| Number of times of response, n(%) |  |  |
| 1 | 0 (0%) | 842 (40%) |
| 2 | 0 (0%) | 1,243 (60%) |
| 3 | 947 (23%) | 0 (0%) |
| 4 | 1,593 (39%) | 0 (0%) |
| 5 | 1,503 (37%) | 0 (0%) |

Notes: Continuous variables are described as median (IQR) and categorical variables are described as number (%). BMI: body mass index, M: million, K: thousand, UCLA: University of California, Los Angeles, SARC-F: a symptom score to predict persons with sarcopenia at risk for poor functional outcomes, MCS: mental component scale, PCS: physical component scale, LTCI: long-term care insurance. ¥3M ($20.6K) is mean annual household income among population aged ≥ 65 in Japan.

**Supplementary Table 6**. Outcome counts and incidence rates of the eligible and ineligible patients

| **Number of outcomes** | **Eligibles N = 4,043** | **Ineligibles N = 2,085** |
| --- | --- | --- |
| Composite outcome | 850 (21.0%) | 862 (41.3%) |
| Loss of independence | 518 (12.8%) | 501 (24.0%) |
| Death | 522 (12.9%) | 637 (30.6%) |
| **Incidence rate of outcomes** |  |  |
| Composite outcome | 42.5 (39.8– 45.2) | 163.0 (152.3–174.3) |
| Loss of independence | 25.9 (23.9–28.1) | 94.8 (86.6–103.4) |
| Death | 24.8 (22.8–26.8) | 95.8 (88.5–103.6) |

**Supplementary Table 7**. Baseline characteristics of the patients stratified by classification of **MCS** trajectories

| **Characteristics** | **Decline, N = 376** | **High stable, N = 3,160** | **Increase, N = 507** |
| --- | --- | --- | --- |
| Age, (years), median (IQR) | 79.7 (76.3, 82.9) | 78.5 (75.6, 82.3) | 79.6 (76.4, 83.3) |
| Sex, n(%) |  |  |  |
| Men | 147 (39%) | 1,475 (47%) | 167 (33%) |
| Women | 229 (61%) | 1,685 (53%) | 340 (67%) |
| BMI, (kg/m^2) , median (IQR) | 22.2 (20.0, 24.7) | 23.1 (21.0, 25.2) | 22.5 (20.4, 25.0) |
| Smoking habit, n(%) |  |  |  |
| Never | 242 (65%) | 1,907 (61%) | 350 (71%) |
| Past | 116 (31%) | 1,023 (33%) | 122 (25%) |
| Current | 14 (3.8%) | 182 (5.8%) | 24 (4.8%) |
| Missing | 4 | 48 | 11 |
| Alcohol habit, n(%) |  |  |  |
| Never | 282 (76%) | 2,186 (70%) | 409 (82%) |
| 1-2 days/week | 27 (7.3%) | 201 (6.4%) | 26 (5.2%) |
| 3-4 days/week | 14 (3.8%) | 193 (6.1%) | 19 (3.8%) |
| 5-6 days/week | 8 (2.2%) | 137 (4.4%) | 10 (2.0%) |
| Everyday | 39 (11%) | 422 (13%) | 36 (7.2%) |
| Missing | 6 | 21 | 7 |
| Unmarried, n(%) | 0 (0%) | 27 (0.9%) | 4 (0.8%) |
| Living alone, n(%) | 63 (17%) | 477 (15%) | 97 (20%) |
| Missing | 8 | 69 | 15 |
| Highest level of education, n(%) |  |  |  |
| Elementary school | 26 (7.1%) | 131 (4.6%) | 23 (5.2%) |
| Junior high school | 177 (48%) | 1,267 (44%) | 236 (54%) |
| High school | 106 (29%) | 973 (34%) | 120 (27%) |
| Junior college | 7 (1.9%) | 69 (2.4%) | 9 (2.0%) |
| Vocational school | 23 (6.3%) | 205 (7.1%) | 24 (5.5%) |
| University | 20 (5.5%) | 187 (6.5%) | 23 (5.2%) |
| Graduate school | 0 (0%) | 5 (0.2%) | 0 (0%) |
| Others | 6 (1.6%) | 39 (1.4%) | 5 (1.1%) |
| Missing | 11 | 284 | 67 |
| Annual household income < ￥3M ($20.6K), n(%) | 81 (22%) | 807 (26%) | 90 (18%) |
| UCLA loneliness scale ≥ 6, n(%) | 132 (36%) | 318 (11%) | 157 (36%) |
| Missing | 9 | 248 | 65 |
| SARC-F ≥ 4, n(%) | 199 (56%) | 585 (20%) | 237 (54%) |
| Missing | 23 | 292 | 69 |
| History of malignant disease, n(%) | 25 (6.6%) | 331 (10%) | 89 (18%) |
| History of myocardial infarction, n(%) | 37 (9.8%) | 402 (13%) | 97 (19%) |
| History of stroke, n(%) | 37 (9.8%) | 434 (14%) | 113 (22%) |
| History of depression, n(%) | 39 (10%) | 438 (14%) | 114 (22%) |
| Diabetes, n(%) | 26 (6.9%) | 373 (12%) | 100 (20%) |
| Baseline MCS score, median (IQR) | 51.2 (47.3, 54.6) | 54.4 (50.5, 55.7) | 42.9 (38.1, 45.9) |
| Baseline PCS score, median (IQR) | 46 (41, 52) | 51 (46, 54) | 44 (39, 49) |
| Baseline LTCI level, n(%) |  |  |  |
| Independent | 347 (92%) | 3,000 (95%) | 430 (85%) |
| Support care level 1 | 13 (3.5%) | 71 (2.2%) | 21 (4.1%) |
| Support care level 2 | 16 (4.3%) | 89 (2.8%) | 56 (11%) |
| Number of times of response, n(%) |  |  |  |
| 3 | 75 (20%) | 700 (22%) | 172 (34%) |
| 4 | 134 (36%) | 1,279 (40%) | 180 (36%) |
| 5 | 167 (44%) | 1,181 (37%) | 155 (31%) |
| Notes: Continuous variables are described as median (IQR) and categorical variables are described as number (%). BMI: body mass index, M: million, K: thousand, UCLA: University of California, Los Angeles, SARC-F: a symptom score to predict persons with sarcopenia at risk for poor functional outcomes, MCS: mental component scale, PCS: physical component scale, LTCI: long-term care insurance. ¥3M ($20.6K) is mean annual household income among population aged ≥ 65 in Japan. | | | |

**Supplementary Table 8.** Baseline characteristics of the patients stratified by classification of **PCS** trajectories

| **Characteristics** | **Low stable, N = 434** | **High stable, N = 3,265** | **Decline, N = 344** |
| --- | --- | --- | --- |
| Age, (years), median (IQR) | 81.7 (78.0, 85.3) | 78.2 (75.5, 81.8) | 81.4 (77.6, 85.1) |
| Sex, n(%) |  |  |  |
| Men | 155 (36%) | 1,483 (45%) | 151 (44%) |
| Women | 279 (64%) | 1,782 (55%) | 193 (56%) |
| BMI, (kg/m^2), median (IQR) | 22.9 (20.5, 25.5) | 22.9 (20.9, 25.0) | 22.8 (20.6, 25.4) |
| Smoking habit, n(%) |  |  |  |
| Never | 284 (67%) | 2,006 (62%) | 209 (62%) |
| Past | 115 (27%) | 1,028 (32%) | 118 (35%) |
| Current | 22 (5.2%) | 186 (5.8%) | 12 (3.5%) |
| Missing | 13 | 45 | 5 |
| Alcohol habit, n(%) |  |  |  |
| Never | 363 (85%) | 2,251 (69%) | 263 (77%) |
| 1-2 days/week | 14 (3.3%) | 222 (6.8%) | 18 (5.3%) |
| 3-4 days/week | 17 (4.0%) | 195 (6.0%) | 14 (4.1%) |
| 5-6 days/week | 6 (1.4%) | 141 (4.4%) | 8 (2.4%) |
| Everyday | 28 (6.5%) | 432 (13%) | 37 (11%) |
| Missing | 6 | 24 | 4 |
| Unmarried, n(%) | 5 (1.2%) | 24 (0.7%) | 2 (0.6%) |
| Living alone, n(%) | 70 (17%) | 498 (16%) | 69 (20%) |
| Missing | 14 | 73 | 5 |
| Highest level of education, n(%) |  |  |  |
| Elementary school | 44 (12%) | 110 (3.7%) | 26 (7.7%) |
| Junior high school | 162 (45%) | 1,355 (45%) | 163 (49%) |
| High school | 94 (26%) | 1,012 (34%) | 93 (28%) |
| Junior college | 9 (2.5%) | 69 (2.3%) | 7 (2.1%) |
| Vocational school | 22 (6.2%) | 210 (7.0%) | 20 (6.0%) |
| University | 21 (5.9%) | 189 (6.3%) | 20 (6.0%) |
| Graduate school | 0 (0%) | 5 (0.2%) | 0 (0%) |
| Others | 5 (1.4%) | 38 (1.3%) | 7 (2.1%) |
| Missing | 77 | 277 | 8 |
| Annual household income < ￥3M ($20.6K), n(%) | 85 (20%) | 825 (25%) | 68 (20%) |
| UCLA loneliness scale ≥ 6, n(%) | 113 (31%) | 397 (13%) | 97 (29%) |
| Missing | 74 | 242 | 6 |
| SARC-F ≥ 4, n(%) | 266 (75%) | 514 (17%) | 241 (73%) |
| Missing | 80 | 289 | 15 |
| History of malignant disease, n(%) | 82 (19%) | 344 (11%) | 19 (5.5%) |
| History of myocardial infarction, n(%) | 78 (18%) | 425 (13%) | 33 (9.6%) |
| History of stroke, n(%) | 97 (22%) | 455 (14%) | 32 (9.3%) |
| History of depression, n(%) | 97 (22%) | 457 (14%) | 37 (11%) |
| Diabetes, n(%) | 79 (18%) | 390 (12%) | 30 (8.7%) |
| Baseline MCS score, median (IQR) | 48.2 (42.3, 53.3) | 53.7 (49.7, 55.0) | 50.0 (45.1, 54.6) |
| Baseline PCS score, median (IQR) | 36 (31, 39) | 51 (47, 54) | 46 (42, 51) |
| Baseline LTCI level, n(%) |  |  |  |
| Independent | 339 (78%) | 3,134 (96%) | 304 (88%) |
| Support care level 1 | 31 (7.1%) | 59 (1.8%) | 15 (4.4%) |
| Support care level 2 | 64 (15%) | 72 (2.2%) | 25 (7.3%) |
| Number of times of response, n(%) |  |  |  |
| 3 | 158 (36%) | 703 (22%) | 86 (25%) |
| 4 | 152 (35%) | 1,327 (41%) | 114 (33%) |
| 5 | 124 (29%) | 1,235 (38%) | 144 (42%) |
| Notes: Continuous variables are described as median (IQR) and categorical variables are described as number (%). BMI: body mass index, M: million, K: thousand, UCLA: University of California, Los Angeles, SARC-F: a symptom score to predict persons with sarcopenia at risk for poor functional outcomes, MCS: mental component scale, PCS: physical component scale, LTCI: long-term care insurance. ¥3M ($20.6K) is mean annual household income among population aged ≥ 65 in Japan. | | | |

**Supplementary Table 9**. Baseline characteristics of the patients stratified by classification of **MCS change score** trajectories

| **Characteristics** | **Decline, N = 475** | **Stable, N = 3,276** | **Increase, N = 292** |
| --- | --- | --- | --- |
| Age, (years), median (IQR) | 79.2 (76.2, 82.8) | 78.6 (75.7, 82.3) | 79.8 (76.5, 84.4) |
| Sex, n(%) |  |  |  |
| Men | 197 (41%) | 1,475 (45%) | 117 (40%) |
| Women | 278 (59%) | 1,801 (55%) | 175 (60%) |
| BMI, (kg/m^2), median (IQR) | 22.8 (20.4, 25.1) | 23.0 (20.9, 25.1) | 23.0 (20.7, 24.7) |
| Smoking habit, n(%) |  |  |  |
| Never | 307 (65%) | 2,009 (62%) | 183 (63%) |
| Past | 143 (30%) | 1,027 (32%) | 91 (31%) |
| Current | 23 (4.9%) | 182 (5.7%) | 15 (5.2%) |
| Missing | 2 | 58 | 3 |
| Alcohol habit, n(%) |  |  |  |
| Never | 345 (73%) | 2,299 (71%) | 233 (80%) |
| 1-2 days/week | 28 (5.9%) | 211 (6.5%) | 15 (5.2%) |
| 3-4 days/week | 26 (5.5%) | 189 (5.8%) | 11 (3.8%) |
| 5-6 days/week | 22 (4.7%) | 130 (4.0%) | 3 (1.0%) |
| Everyday | 50 (11%) | 418 (13%) | 29 (10.0%) |
| Missing | 4 | 29 | 1 |
| Unmarried, n(%) | 3 (0.6%) | 25 (0.8%) | 3 (1.0%) |
| Living alone, n(%) | 67 (15%) | 504 (16%) | 66 (23%) |
| Missing | 15 | 72 | 5 |
| Highest level of education, n(%) |  |  |  |
| Elementary school | 35 (7.6%) | 121 (4.1%) | 24 (8.5%) |
| Junior high school | 205 (45%) | 1,350 (46%) | 125 (44%) |
| High school | 152 (33%) | 955 (33%) | 92 (32%) |
| Junior college | 9 (2.0%) | 69 (2.3%) | 7 (2.5%) |
| Vocational school | 26 (5.7%) | 209 (7.1%) | 17 (6.0%) |
| University | 25 (5.4%) | 192 (6.5%) | 13 (4.6%) |
| Graduate school | 0 (0%) | 5 (0.2%) | 0 (0%) |
| Others | 8 (1.7%) | 36 (1.2%) | 6 (2.1%) |
| Missing | 15 | 339 | 8 |
| Annual household income < ￥3M ($20.6K), n(%) | 102 (21%) | 807 (25%) | 69 (24%) |
| UCLA loneliness scale ≥ 6, n(%) | 135 (29%) | 418 (14%) | 54 (19%) |
| Missing | 12 | 305 | 5 |
| SARC-F ≥ 4, n(%) | 218 (48%) | 685 (23%) | 118 (41%) |
| Missing | 23 | 354 | 7 |
| History of malignant disease, n(%) | 30 (6.3%) | 400 (12%) | 15 (5.1%) |
| History of myocardial infarction, n(%) | 43 (9.1%) | 470 (14%) | 23 (7.9%) |
| History of stroke, n(%) | 44 (9.3%) | 513 (16%) | 27 (9.2%) |
| History of depression, n(%) | 44 (9.3%) | 522 (16%) | 25 (8.6%) |
| Diabetes, n(%) | 33 (6.9%) | 441 (13%) | 25 (8.6%) |
| Baseline MCS score, median (IQR) | 54.8 (52.2, 56.7) | 53.2 (49.0, 54.9) | 42.9 (38.0, 46.3) |
| Baseline PCS score, median (IQR) | 46 (41, 51) | 51 (45, 54) | 47 (39, 53) |
| Baseline LTCI level, n(%) |  |  |  |
| Independent | 440 (93%) | 3,075 (94%) | 262 (90%) |
| Support care level 1 | 17 (3.6%) | 78 (2.4%) | 10 (3.4%) |
| Support care level 2 | 18 (3.8%) | 123 (3.8%) | 20 (6.8%) |
| Number of times of response, n(%) |  |  |  |
| 3 | 99 (21%) | 787 (24%) | 61 (21%) |
| 4 | 187 (39%) | 1,293 (39%) | 113 (39%) |
| 5 | 189 (40%) | 1,196 (37%) | 118 (40%) |
| Notes: Continuous variables are described as median (IQR) and categorical variables are described as number (%). BMI: body mass index, M: million, K: thousand, UCLA: University of California, Los Angeles, SARC-F: a symptom score to predict persons with sarcopenia at risk for poor functional outcomes, MCS: mental component scale, PCS: physical component scale, LTCI: long-term care insurance. ¥3M ($20.6K) is mean annual household income among population aged ≥ 65 in Japan. | | | |

**Supplementary Table 10**. Baseline characteristics of the patients stratified by classification of **PCS change score** trajectories

| **Characteristics** | **Decline, N = 442** | **Stable N = 3,292** | **Increase, N = 309** |
| --- | --- | --- | --- |
| Age, (years), median (IQR) | 80.1 (76.8, 84.5) | 78.6 (75.7, 82.3) | 78.2 (75.2, 81.7) |
| Sex, n(%) |  |  |  |
| Men | 191 (43%) | 1,472 (45%) | 126 (41%) |
| Women | 251 (57%) | 1,820 (55%) | 183 (59%) |
| BMI, (kg/m^2), median (IQR) | 22.9 (20.5, 25.5) | 22.9 (20.9, 25.0) | 22.9 (21.0, 25.5) |
| Smoking habit, n(%) |  |  |  |
| Never | 277 (64%) | 2,024 (62%) | 198 (65%) |
| Past | 141 (32%) | 1,028 (32%) | 92 (30%) |
| Current | 18 (4.1%) | 189 (5.8%) | 13 (4.3%) |
| Missing | 6 | 51 | 6 |
| Alcohol habit, n(%) |  |  |  |
| Never | 327 (75%) | 2,329 (71%) | 221 (72%) |
| 1-2 days/week | 20 (4.6%) | 213 (6.5%) | 21 (6.9%) |
| 3-4 days/week | 18 (4.1%) | 188 (5.8%) | 20 (6.5%) |
| 5-6 days/week | 17 (3.9%) | 126 (3.9%) | 12 (3.9%) |
| Everyday | 56 (13%) | 409 (13%) | 32 (10%) |
| Missing | 4 | 27 | 3 |
| Unmarried, n(%) | 2 (0.5%) | 28 (0.9%) | 1 (0.3%) |
| Living alone, n(%) | 73 (17%) | 510 (16%) | 54 (18%) |
| Missing | 6 | 78 | 8 |
| Highest level of education, n(%) |  |  |  |
| Elementary school | 32 (7.4%) | 134 (4.5%) | 14 (4.7%) |
| Junior high school | 220 (51%) | 1,314 (44%) | 146 (49%) |
| High school | 113 (26%) | 1,000 (34%) | 86 (29%) |
| Junior college | 10 (2.3%) | 66 (2.2%) | 9 (3.0%) |
| Vocational school | 25 (5.8%) | 211 (7.1%) | 16 (5.4%) |
| University | 26 (6.0%) | 183 (6.2%) | 21 (7.1%) |
| Graduate school | 0 (0%) | 5 (0.2%) | 0 (0%) |
| Others | 5 (1.2%) | 41 (1.4%) | 4 (1.4%) |
| Missing | 11 | 338 | 13 |
| Annual household income < ￥3M ($20.6K), n(%) | 87 (20%) | 813 (25%) | 78 (25%) |
| UCLA loneliness scale ≥ 6, n(%) | 102 (24%) | 451 (15%) | 54 (18%) |
| Missing | 9 | 305 | 8 |
| SARC-F ≥ 4, n(%) | 226 (53%) | 718 (24%) | 77 (26%) |
| Missing | 19 | 350 | 15 |
| History of malignant disease, n(%) | 26 (5.9%) | 398 (12%) | 21 (6.8%) |
| History of myocardial infarction, n(%) | 41 (9.3%) | 467 (14%) | 28 (9.1%) |
| History of stroke, n(%) | 42 (9.5%) | 511 (16%) | 31 (10%) |
| History of depression, n(%) | 47 (11%) | 513 (16%) | 31 (10%) |
| Diabetes, n(%) | 42 (9.5%) | 432 (13%) | 25 (8.1%) |
| Baseline MCS score, median (IQR) | 51.3 (46.0, 54.6) | 53.2 (48.8, 55.0) | 52.7 (47.2, 56.8) |
| Baseline PCS score, median (IQR) | 51 (46, 55) | 50 (45, 54) | 41 (34, 45) |
| Baseline LTCI level, n(%) |  |  |  |
| Independent | 409 (93%) | 3,092 (94%) | 276 (89%) |
| Support care level 1 | 15 (3.4%) | 79 (2.4%) | 11 (3.6%) |
| Support care level 2 | 18 (4.1%) | 121 (3.7%) | 22 (7.1%) |
| Number of times of response, n(%) |  |  |  |
| 3 | 99 (22%) | 786 (24%) | 62 (20%) |
| 4 | 157 (36%) | 1,310 (40%) | 126 (41%) |
| 5 | 186 (42%) | 1,196 (36%) | 121 (39%) |
| Notes: Continuous variables are described as median (IQR) and categorical variables are described as number (%). BMI: body mass index, M: million, K: thousand, UCLA: University of California, Los Angeles, SARC-F: a symptom score to predict persons with sarcopenia at risk for poor functional outcomes, MCS: mental component scale, PCS: physical component scale, LTCI: long-term care insurance. ¥3M ($20.6K) is mean annual household income among population aged ≥ 65 in Japan. | | | |

**Supplementary Figure 5**. Kaplan-Meier survival estimates for composite outcome–free survival in each MCS trajectory

**
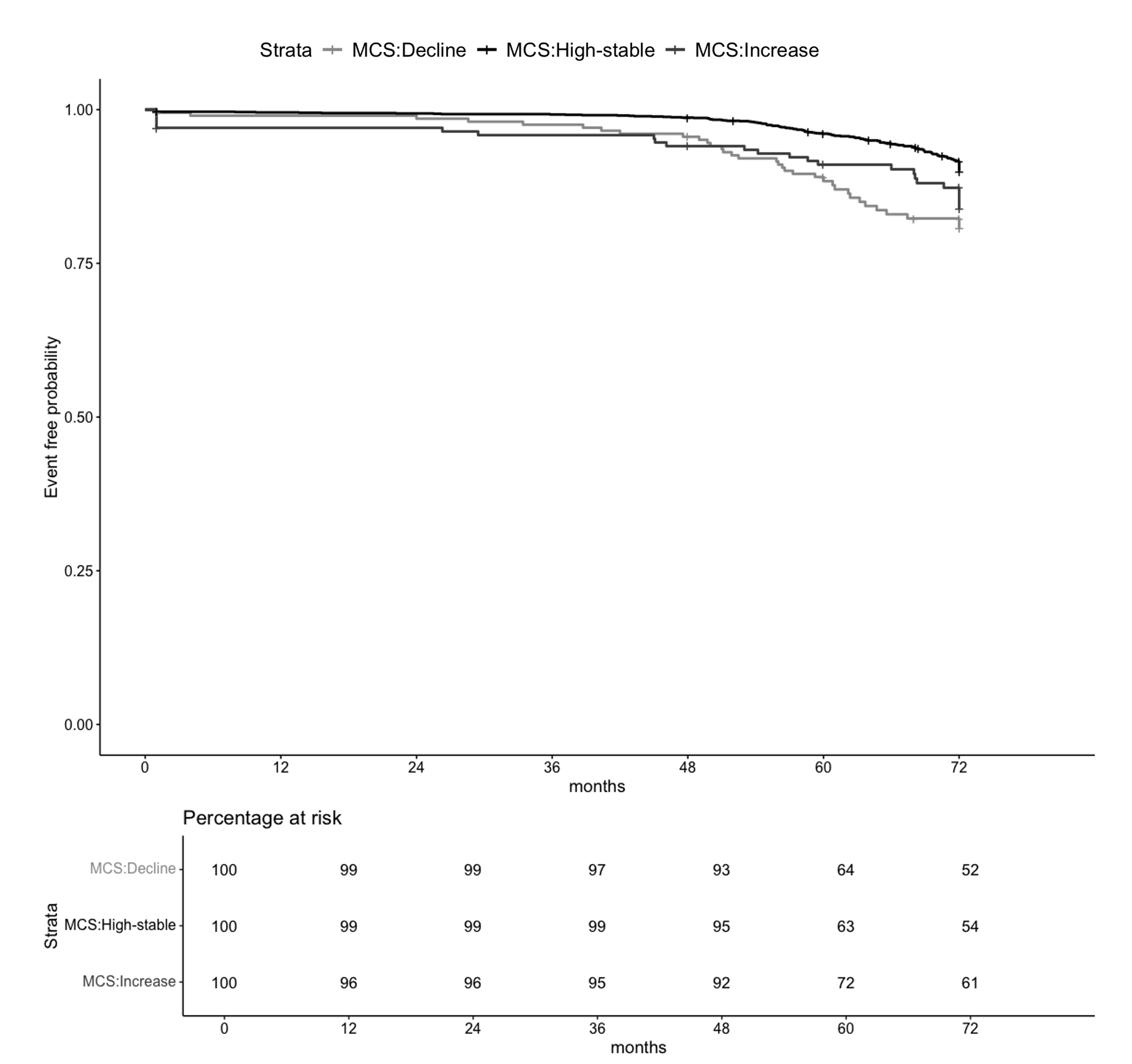
**

Notes, MCS: mental component scale,

The composite outcome is defined as loss of independence (LOI) or death from any cause.

**Supplementary Figure 6**. Kaplan-Meier survival estimates for composite outcome–free survival in each PCS trajectory

**
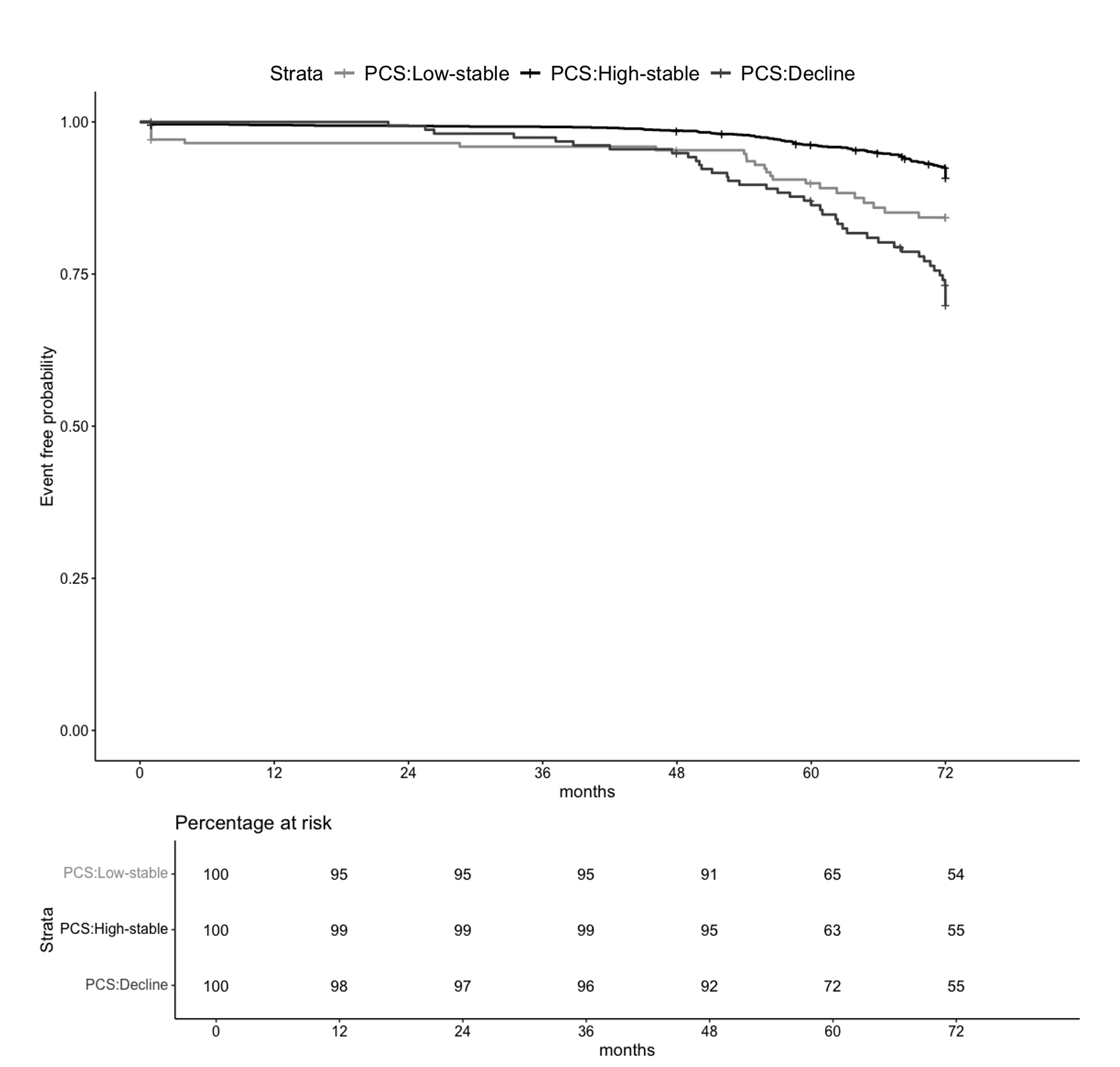
**

Notes, PCS: physical component scale,

The composite outcome is defined as loss of independence (LOI) or death from any cause.

**Supplementary Figure 7**. Kaplan-Meier survival estimates for composite outcome–free survival in each MCS change score trajectory

**
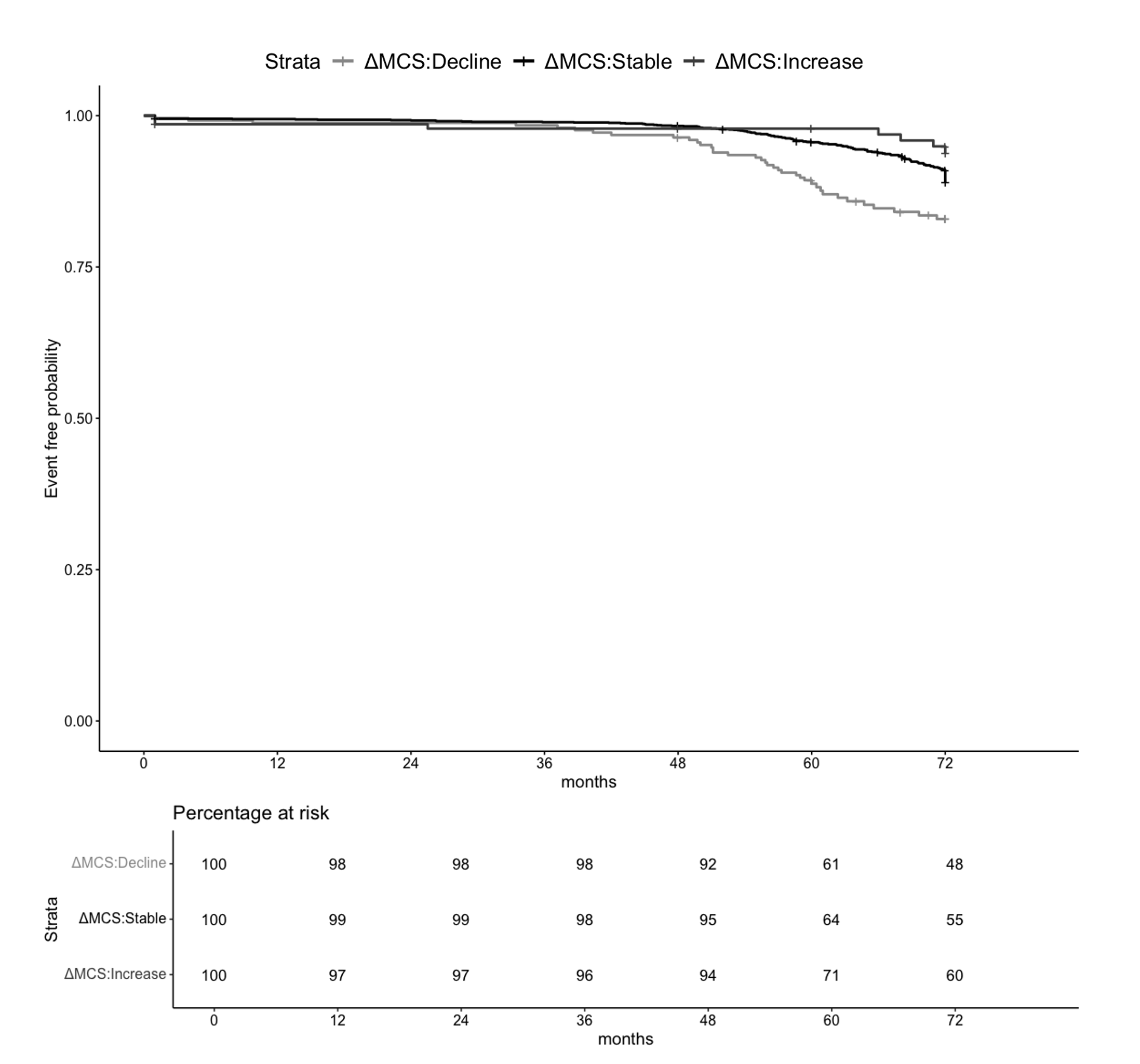
**

Notes, ΔMCS: change score of the Mental Component scale,

The composite outcome is defined as loss of independence (LOI) or death from any cause.

**Supplementary Figure 8**. Kaplan-Meier survival estimates for composite outcome–free survival in each PCS change score trajectory

**
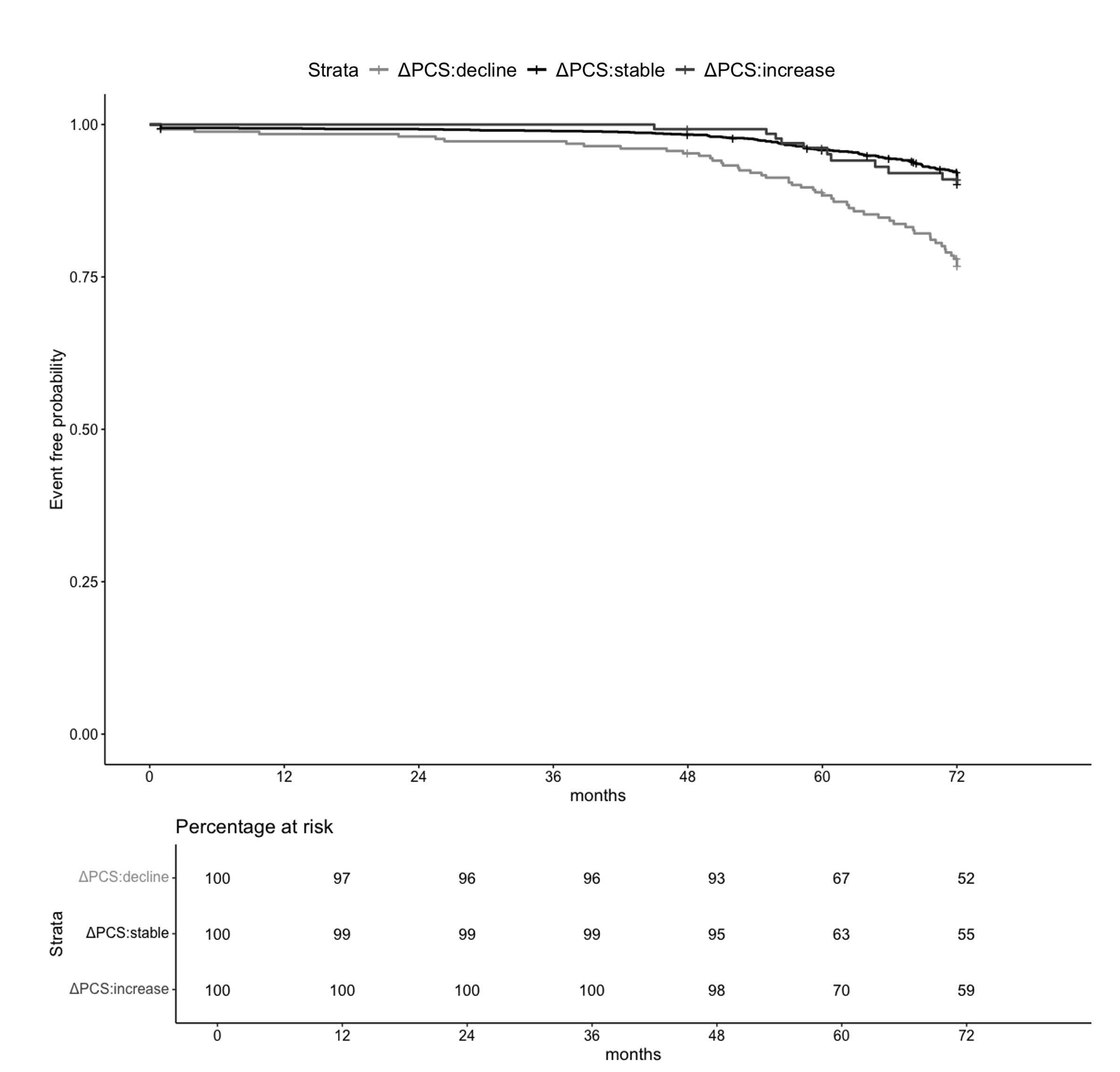
**

Notes, ΔPCS: change score of the Physicall Component scale,

The composite outcome is defined as loss of independence (LOI) or death from any cause.

**Supplementary Figure 9**. Kaplan-Meier survival estimates for composite outcome–free survival stratified by MCS and PCS trajectories


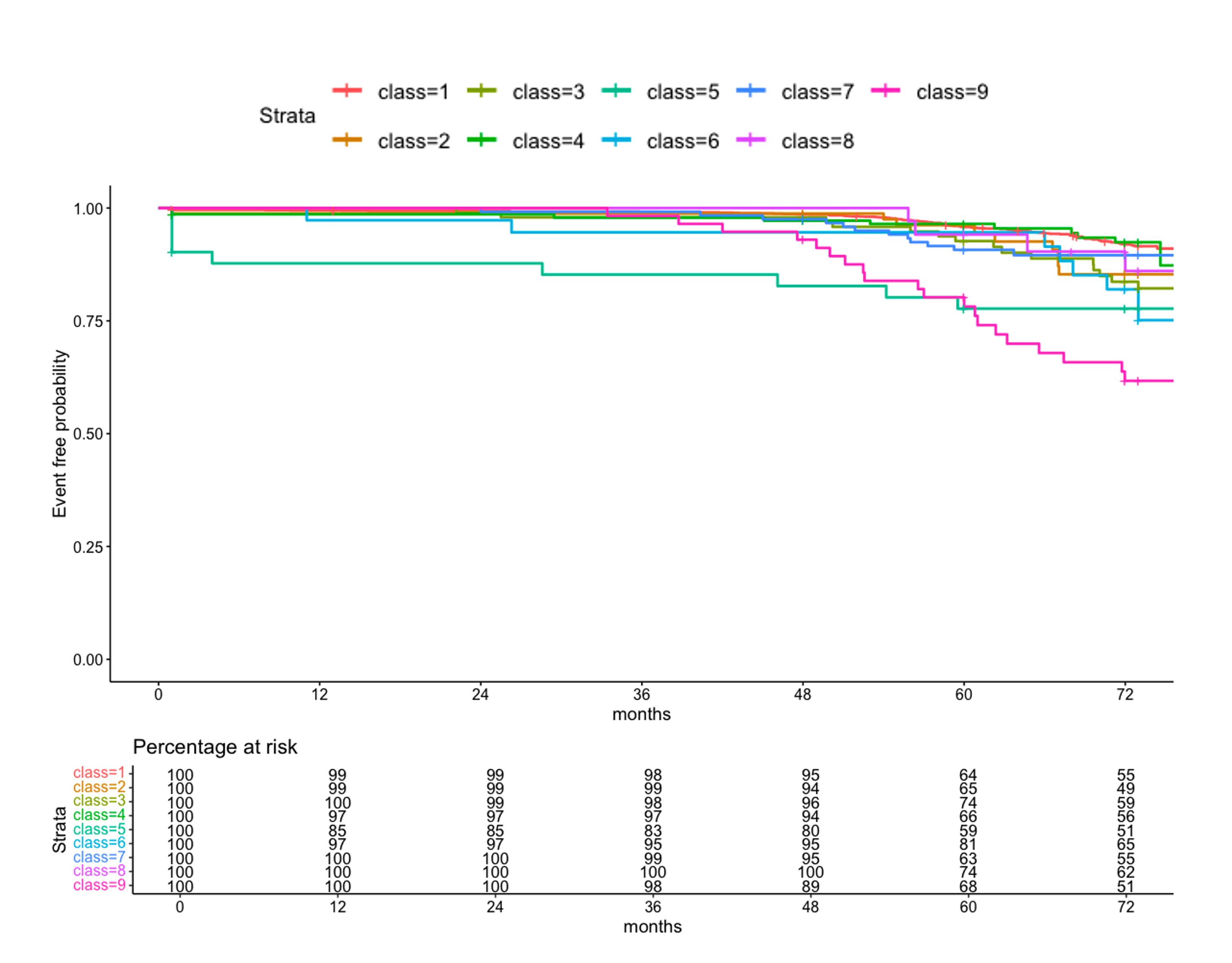


Notes, MCS: mental component scale, PCS: physical component scale,

class1 (MCS: high stable & PCS: high stable), class 2 (MCS: high stable & PCS: low stable), class 3 (MCS: high stable & PCS: decline),

class 4 (MCS: increase & PCS: high stable), class 5 (MCS: increase & PCS: low stable), class 6 (MCS: increase & PCS: decline),

class 7 (MCS: decline & PCS: high stable), class 8 (MCS: decline & PCS: low stable), class 9 (MCS: decline & PCS: decline)

The composite outcome is defined as loss of independence (LOI) or death from any cause.

**Supplementary Figure 10**. Kaplan-Meier survival estimates for composite outcome–free survival stratified by MCS and PCS change score trajectories

**
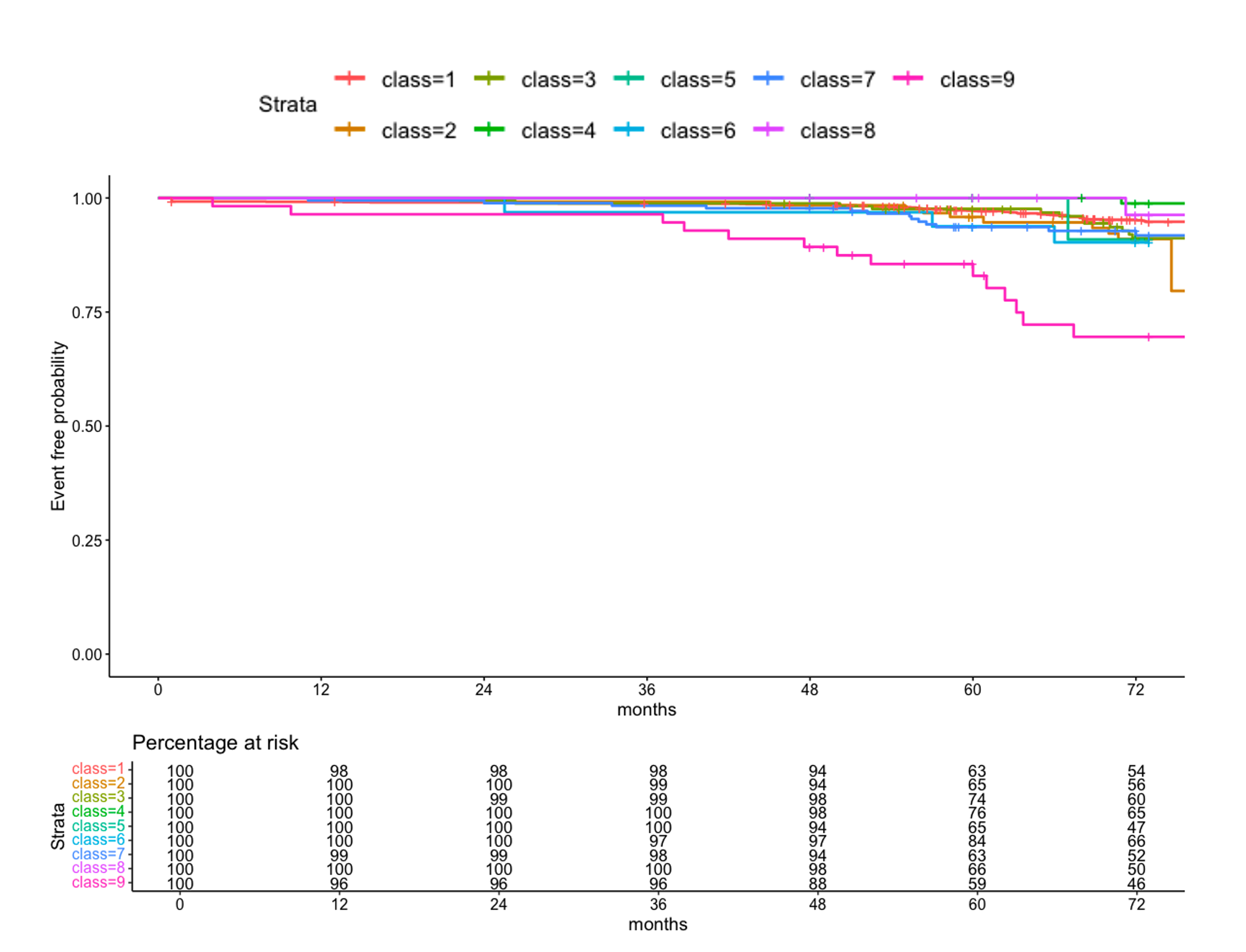
**

Notes, Mc: mental component scale change score, Pc: physical component scale change score,

class1 (Mc stable & Pc: stable), class 2 (Mc stable & Pc: increase), class 3 (Mc stable & Pc: decline),

class 4 (Mc: increase & Pc: stable), class 5 (Mc: increase & Pc: increase), class 6 (Mc: increase & Pc: decline),

class 7 (Mc: decline & Pc: stable), class 8 (Mc: decline & Pc: increase), class 9 (Mc: decline & Pc: decline)

The composite outcome is defined as loss of independence (LOI) or death from any cause.

**Supplementary Table 11.** Association of outcomes occurring after 60 months with trajectory patterns of HRQOL (Sensitivity analysis 1)

|  | MCS trajectory class |  |  |
| --- | --- | --- | --- |
|  | **Increase** | **High stable** | **Decline** |
| Composite |  |  |  |
| Adjusted hazard ratio (95% CI) | 1.11 (0.58-2.15) | 1 [Reference] | **1.63 (1.06-2.51)*** |
| Loss of independence |  |  |  |
| Adjusted hazard ratio (95% CI) | 1.15 (0.49-2.69) | 1 [Reference] | **2.15(1.25-3.69)*** |
| Death |  |  |  |
| Adjusted hazard ratio (95% CI) | 1.71 (0.85-3.45) | 1 [Reference] | 1.47 (0.86-2.52) |
|  | **PCS trajectory class** |  |  |
|  | **Low stable** | **High stable** | **Decline** |
| Composite |  |  |  |
| Adjusted hazard ratio (95% CI) | 1.21 (0.82-2.44) | 1 [Reference] | **2.32 (1.57-3.45)*** |
| Loss of independence |  |  |  |
| Adjusted hazard ratio (95% CI) | 0.97 (0.38-2.51) | 1 [Reference] | **2.34 (1.38-3.96)*** |
| Death |  |  |  |
| Adjusted hazard ratio (95% CI) | **2.34 (1.07-5.12)*** | 1 [Reference] | **3.06 (1.94-4.82)*** |
|  | **MCS change score trajectory** class |  |  |
|  | **Decline** | **Stable** | **Increase** |
| Composite |  |  |  |
| Adjusted hazard ratio (95% CI) | **1.51(1.00-2.28)*** | 1 [Reference] | **0.32 (0.13-0.78)*** |
| Loss of independence |  |  |  |
| Adjusted hazard ratio (95% CI) | 1.63 (0.93-2.84) | 1 [Reference] | **0.30 (0.10-0.91)*** |
| Death |  |  |  |
| Adjusted hazard ratio (95% CI) | 1.78 (1.12-2.84) | 1 [Reference] | **0.36 (0.14-0.98)*** |
|  | **PCS change score trajectory** |  |  |
|  | **Decline** | **Stable** | **Increase** |
| Composite |  |  |  |
| Adjusted hazard ratio (95% CI) | **2.25 (1.57-3.23)*** | 1 [Reference] | 1.01 (0.56-1.85) |
| Loss of independence |  |  |  |
| Hazard ratio (95% CI) | **2.11 (1.28-3.46)*** | 1 [Reference] | 1.05 (0.48-2.31) |
| Death |  |  |  |
| Adjusted hazard ratio (95% CI) | **2.58 (1.71-3.90)*** | 1 [Reference] | 0.53 (0.22-1.29) |

Notes, 95% CI: 95% confidence interval, *: *p* value < 0.05

Adjusted for sex, age, body mass index, smoking habits, alcohol intake, marital status, living alone, highest level of education, annual household income, UCLA loneliness scale ≥ 6, SARC-F ≥ 4, history of being diagnosed with malignant disease, myocardial infarction, stroke, depression, and diabetes, baseline MCS and PCS in SF-8, The composite outcome is defined as loss of independence (LOI) or death from any cause.

**Supplementary Table 12**. Association of outcomes with trajectory patterns of HRQOL among participants who were independent at baseline (Sensitivity analysis 2)

|  | MCS trajectory class |  |  |
| --- | --- | --- | --- |
|  | **Increase** | **High stable** | **Decline** |
| Composite |  |  |  |
| Adjusted hazard ratio (95% CI) | 1.19 (0.67-2.10) | 1 [Reference] | **1.50 (1.00-2.25)*** |
| Loss of independence |  |  |  |
| Adjusted hazard ratio (95% CI) | 1.19 (0.57-2.47) | 1 [Reference] | **2.03 (1.24-3.33)*** |
| Death |  |  |  |
| Adjusted hazard ratio (95% CI) | 1.20 (0.57-2.56) | 1 [Reference] | 1.24 (0.71-2.15) |
|  | **PCS trajectory class** |  |  |
|  | **Low stable** | **High stable** | **Decline** |
| Composite |  |  |  |
| Adjusted hazard ratio (95% CI) | 1.17 (0.62-2.21) | 1 [Reference] | **1.78 (1.19-2.66)*** |
| Loss of independence |  |  |  |
| Adjusted hazard ratio (95% CI) | 1.19 (0.52-2.72) | 1 [Reference] | **1.87 (1.12-3.10)*** |
| Death |  |  |  |
| Adjusted hazard ratio (95% CI) | 1.60 (0.69-3.74) | 1 [Reference] | **1.98 (1.18-3.32)*** |
|  | **MCS change score trajectory** class |  |  |
|  | **Decline** | **Stable** | **Increase** |
| Composite |  |  |  |
| Adjusted hazard ratio (95% CI) | 1.41 (0.95-2.08) | 1 [Reference] | **0.20 (0.08-0.52)*** |
| Loss of independence |  |  |  |
| Adjusted hazard ratio (95% CI) | **1.73 (1.05-2.84)*** | 1 [Reference] | **0.23 (0.08-0.66)*** |
| Death |  |  |  |
| Adjusted hazard ratio (95% CI) | 1.30 (0.78-2.16) | 1 [Reference] | 0.36 (0.12-1.05) |
|  | **PCS change score trajectory** |  |  |
|  | **Decline** | **Stable** | **Increase** |
| Composite |  |  |  |
| Adjusted hazard ratio (95% CI) | **1.75 (1.21-2.51)*** | 1 [Reference] | 1.15 (0.64-2.08) |
| Loss of independence |  |  |  |
| Hazard ratio (95% CI) | **1.64 (1.02-2.65)*** | 1 [Reference] | 1.31 (0.62-2.80) |
| Death |  |  |  |
| Adjusted hazard ratio (95% CI) | **1.93 (1.22-3.06)*** | 1 [Reference] | 0.80 (0.32-2.01) |

Notes, 95% CI: 95% confidence interval, *: *p* value < 0.05

Adjusted for sex, age, body mass index, smoking habits, alcohol intake, marital status, living alone, highest level of education, annual household income, UCLA loneliness scale ≥ 6, SARC-F ≥ 4, history of being diagnosed with malignant disease, myocardial infarction, stroke, depression, and diabetes, baseline MCS and PCS in SF-8, The composite outcome is defined as loss of independence (LOI) or death from any cause.
